# Screening of microRNAs and target genes involved in *Sclerotinia sclerotiorum* (Lib.) infection in *Brassica napus* L.

**DOI:** 10.1186/s12870-023-04501-7

**Published:** 2023-10-09

**Authors:** Ling Xie, Hongju Jian, Haoxi Dai, Youhong Yang, Yiling Liu, Lijuan Wei, Min Tan, Jiana Li, Liezhao Liu

**Affiliations:** College of Agronomy and Biotechnology, Southwest University, Chongqing, 400715 China

**Keywords:** *Brassica napus* L., *Sclerotinia sclerotiorum*, Differentially expressed miRNAs, Target genes, miR156

## Abstract

**Background:**

Rapeseed (*Brassica napus* L.) is the third largest source of vegetable oil in the world, and *Sclerotinia sclerotiorum* (Lib.) is a major soil-borne fungal plant pathogen that infects more than 400 plant species, including *B. napus*. *Sclerotinia* stem rot caused an annual loss of 10 − 20% in rapeseed yield. Exploring the molecular mechanisms in response to *S. sclerotiorum* infection in *B. napus* is beneficial for breeding and cultivation of resistant varieties. To gain a better understanding of the mechanisms regarding *B. napus* tolerance to *Sclerotinia* stem rot, we employed a miRNAome sequencing approach and comprehensively investigated global miRNA expression profile among five relatively resistant lines and five susceptible lines of oilseed at 0, 24, and 48 h post-inoculation.

**Results:**

In this study, a total of 40 known and 1105 novel miRNAs were differentially expressed after *S. sclerotiorum* infection, including miR156, miR6028, miR394, miR390, miR395, miR166, miR171, miR167, miR164, and miR172. Furthermore, 8,523 genes were predicted as targets for these differentially expressed miRNAs. These target genes were mainly associated with disease resistance (*R*) genes, signal transduction, transcription factors, and hormones. Constitutively expressing miR156b (OX156b) plants strengthened Arabidopsis resistance against *S. sclerotiorum* accompanied by smaller necrotic lesions, whereas blocking miR156 expression in Arabidopsis (MIM156) led to greater susceptibility to *S. sclerotiorum* disease, associated with extensive cell death of necrotic lesions.

**Conclusions:**

This study reveals the distinct difference in miRNA profiling between the relatively resistant lines and susceptible lines of *B. napus* in response to *S. sclerotiorum*. The identified differentially expressed miRNAs related to *sclerotinia* stem rot resistance are involved in regulating resistance to *S. sclerotiorum* in rapeseed by targeting genes related to *R* genes, signal transduction, transcription factors, and hormones. miR156 positively modulates the resistance to *S. sclerotiorum* infection by restricting colonization of *S. sclerotiorum* mycelia. This study provides a broad view of miRNA expression changes after *S. sclerotiorum* infection in oilseed and is the first to elucidate the function and mechanism underlying the miR156 response to *S. sclerotiorum* infection in oilseed rape.

**Supplementary Information:**

The online version contains supplementary material available at 10.1186/s12870-023-04501-7.

## Backgrounds

*Sclerotinia sclerotiorum* (Lib.) is a major soil-borne fungal plant pathogen that infects more than 400 plant species and causes root, crown, and stem rot on various plant hosts [1]. As necrotrophic phytopathogenic fungus, *S. sclerotiorum* obtains nutrients from plants by infecting and killing host cells and destroying host tissue, causing significant yield losses and economic damage to many economically important crops, including oilseed rape [2]. Fungicides and biocontrol agents have been used to restrain this pathogen in the past. However, this method pollutes the environment. Thus, screening and breeding of relatively resistant oilseed rape cultivars is urgently needed, but information about how oilseed rape responds to *S*. *sclerotiorum* infection is lacking, even though much effort has been made.

As endogenous and noncoding RNAs, miRNAs play key roles in diverse biological processes by binding to the 3′ untranslated regions of messenger RNAs [3]. miRNAs have been reported as key components in various biological processes, such as development [4], organ formation [5], and many environmental stresses [6]. In addition, several studies have suggested that miRNAs play key roles in pathogen attacks [7, 8]. In *Arabidopsis thaliana*, miR393 and miR396 are involved in the defence response to *Pseudomonas syringae* pv. tomato (*Pst*) and cyst nematode, respectively [7, 9]. In response to *Pst* infection, miR393 negatively regulates messenger RNAs for auxin receptors, transport inhibitor response 1 (TIR1), auxin signalling F-box protein 2 (AFB2), and AFB3 and positively regulates the defence response through auxin signalling [7]. Moreover, miR393 plays a key role in the regulation of the glucosinolate pathway, which participates in plant responses to pathogens [10]. As a positive regulator in cyst nematode infection, miR396 suppressed the target gene encoding growth regulating factor 1/3 (*GRF1*/*3*) [9]. In *Brassica rapa*, miR158 and miR1885 play key roles in the response to turnip mosaic virus infection by suppressing the disease resistance protein gene nucleotide-binding site leucine-rich repeat [11]. In tobacco, miR6019 and miR6020 contributed to the resistance of tobacco mosaic virus by guiding the incision of N genes [12]. In rice, small RNA profiling of resistant and susceptible lines after *Magnaporthe oryzae* (*M. oryzae*) infection revealed that miR156, miR160, miR169, and miR164 were induced, miR394 and miR396 were downregulated in the resistant lines but were not observed in the susceptible lines. In addition, overexpression of miR160a or miR398b in susceptible rice cultivars could enhance rice resistance to *M. oryzae* [13].

miR156 is one of the most abundant and highly conserved miRNAs in plants and has been extensively investigated. miR156 has been reported to be significantly upregulated after infection with fungal phytopathogens such as *Botrytis cinereal* (*B. cinereal*), *Dothiorella gregari*a (*D. gregaria*), and *S. sclerotiorum* in Arabidopsis, tomato, *populus*, and oilseed rape [14–18]. Furthermore, a negative effect of miR156-regulated *Squamosa promoter binding protein-like* (*SPL9*) on plant resistance for *Helicoverpa armigera* and *Plutella xylostella* was observed in Arabidopsis [19]. However, many studies have also proven that miR156 negatively regulates other fungal and bacterial diseases and insect resistance. Downregulation of miR156 and overexpression of *OsSPL7* and *AtSPL9* enhanced disease resistance against bacterial blight and *Pst* DC3000 in rice and Arabidopsis, respectively [20, 21]. Silencing of miR156 increased the expression of defence-related genes and enhanced rice blast and brown planthopper resistance in rice [22, 23]. These results show that the specific functions of miR156/SPL networks are not highly conserved when exposed to biotic stresses in different species. The study on the regulation of *S. sclerotiorum* resistance by miRNA and miR156 in oilseed rape only remained at the transcriptome level, so the role of miR156 in response to *S. sclerotiorum* remains unclear.

Identification of pathogen-responsive miRNAs and their targets will help elucidate the complex miRNA-mediated regulatory networks behind the plant response to *S. sclerotiorum* infection. However, little is known about the regulation of *S. sclerotiorum* resistance by small RNAs. Here, five relatively resistant lines (R-lines) and five relatively susceptible lines (S-lines) were used as materials, and samples at three time points (0 h, 24 h, and 48 h) were tested to investigate miRNAs involved in *S. sclerotiorum* infection in oilseed rape and explore the resistance mechanism. The results of transgenic functional verification showed that miR156 positively regulates rapeseed resistance to *S. sclerotiorum*. This study added more useful information on the molecular mechanisms in response to *S. sclerotiorum* infection in oilseed rape.

## Results

### Deep sequencing analysis of sRNAs in oilseed rape

**Fig. 1:**
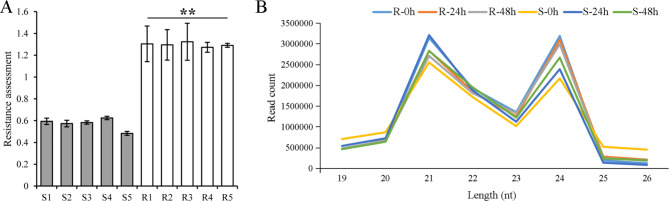
The resistance assessment (RA) of 5 resistant (R) and 5 susceptible (S) *B. napus* winter-type accessions inoculated with *S. sclerotiorum* (**A**) and length distribution of small RNAs obtained from the six libraries of oilseed rape in this study (**B**). Values are the means ± SDs from ten replicates. The significant differences from S are indicated (Student’s *t* test: **. *P* < 0.01)

The resistance assessment (RA) of 5 resistant (R) and 5 susceptible (S) *B. napus* winter-type accessions inoculated with *S. sclerotiorum* showed that the RA of the resistant accessions was obviously higher than susceptible accessions (Fig. 1A). In total, 12,340,495 (R-0 h), 11,926,637 (R-24 h), 11,623,972 (R-48 h), 11,672,636 (S-0 h), 11,547,239 (S-24 h), and 11,528,861 (S-48 h) raw reads were obtained in six oilseed rape samples (Table S3). After filtration, 11,639,549 (R-0 h), 11,539,673 (R-24 h), 11,031,935 (R-48 h), 11,317,740 (S-0 h), 10,607,203 (S-24 h), and 10,909,522 (S-48 h) clean reads corresponding to 3,957,503 (34.0%), 3,716,475 (32.2%), 3,708,260 (33.6%), 2,541,507 (22.5%), 3,403,808 (32.1%), and 3,212,007 (29.4%) unique clean reads were obtained for the R-0 h, R-24 h, R-48 h, S-0 h, S-24 h, and S-48 h libraries, respectively (Table S3). For identification of the sequence categories, all clean reads were queried against the *Brassica napus* L. genome, Rfam and miRBase v.22, and 11 annotated categories were classified (Table S1). The length distribution of the total sRNA reads showed that 19–26 nt in length accounted for the majority of reads in each library (Fig. 1). Among them, 21 and 24 nt reads were the top two most abundant. Interestingly, 24 nt was the most abundant in the R accessions, while 21 nt was the most abundant in the S accessions (Fig. 1B).

### Identification of known and novel miRNAs in *B. napus*

**Fig. 2:**
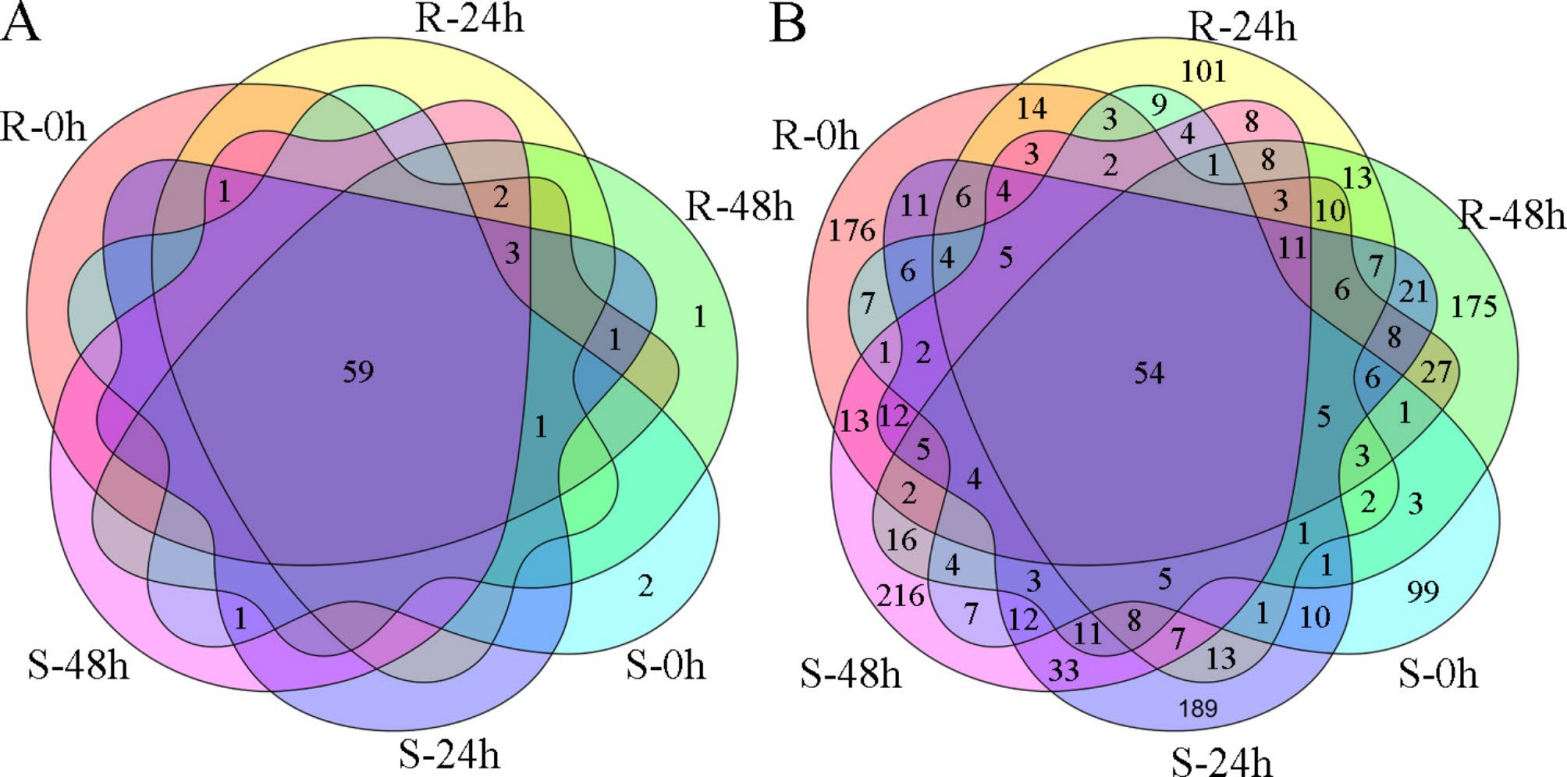
Venn diagrams of known miRNAs (**A**) and novel miRNAs (**B**) in six libraries

To identify known miRNAs from six libraries, we mapped all unique reads to the known plant miRNAs in the miRBase v. 22.0 database. In total, 72 known miRNAs belonging to 29 miRNA families were identified in six libraries (Table S4). Among these 29 families, miR156 and miR171 had the most members, containing seven members, followed by miR166 and miR395, which both contained six members; 15 miRNA families contained only one miRNA member (Table S4). Among them, 59 (81.9%) miRNAs were detected in all six libraries (Fig. 2A). For the total reads of each miRNA, 38 (52.8%) had more than 100 reads, 11 (15.3%) had more than 1000 reads, and only 8 (11.1%) had more than 10,000 reads. Among them, miR159 had the highest expression level in each library, followed by miR403 in R accessions and miR396a in S accessions. Interestingly, miR6028 was only expressed in S accessions (Table S4).

After identification of the known miRNAs, the “unannotated” sequences were used to predict novel miRNAs in *B. napus*. In total, 1,402 novel miRNAs from six libraries were identified (Table S5). Among them, only 54 (3.9%) miRNAs were detected in all six libraries (Fig. 2B). For the total reads of each miRNA, 46 (3.3%) had more than 100 reads, and only 16 (1.1%) had more than 1000 reads; novel_mir_1, novel_mir_34, novel_mir_35, and novel_mir_62 had more than 10,000 reads (Table S5). In addition, the majority of reads were 20–23 nt in length, 23 nt and 21 nt were peaks in all six libraries (Fig. S1), which was different from that in known miRNAs detected in this study. Interestingly, the base of the first position of the 5′ end in all six libraries was mainly the uracil nucleotide (Fig. S2).

### Differentially expressed miRNAs in *B. napus* after *S. sclerotiorum* infection

**Fig. 3:**
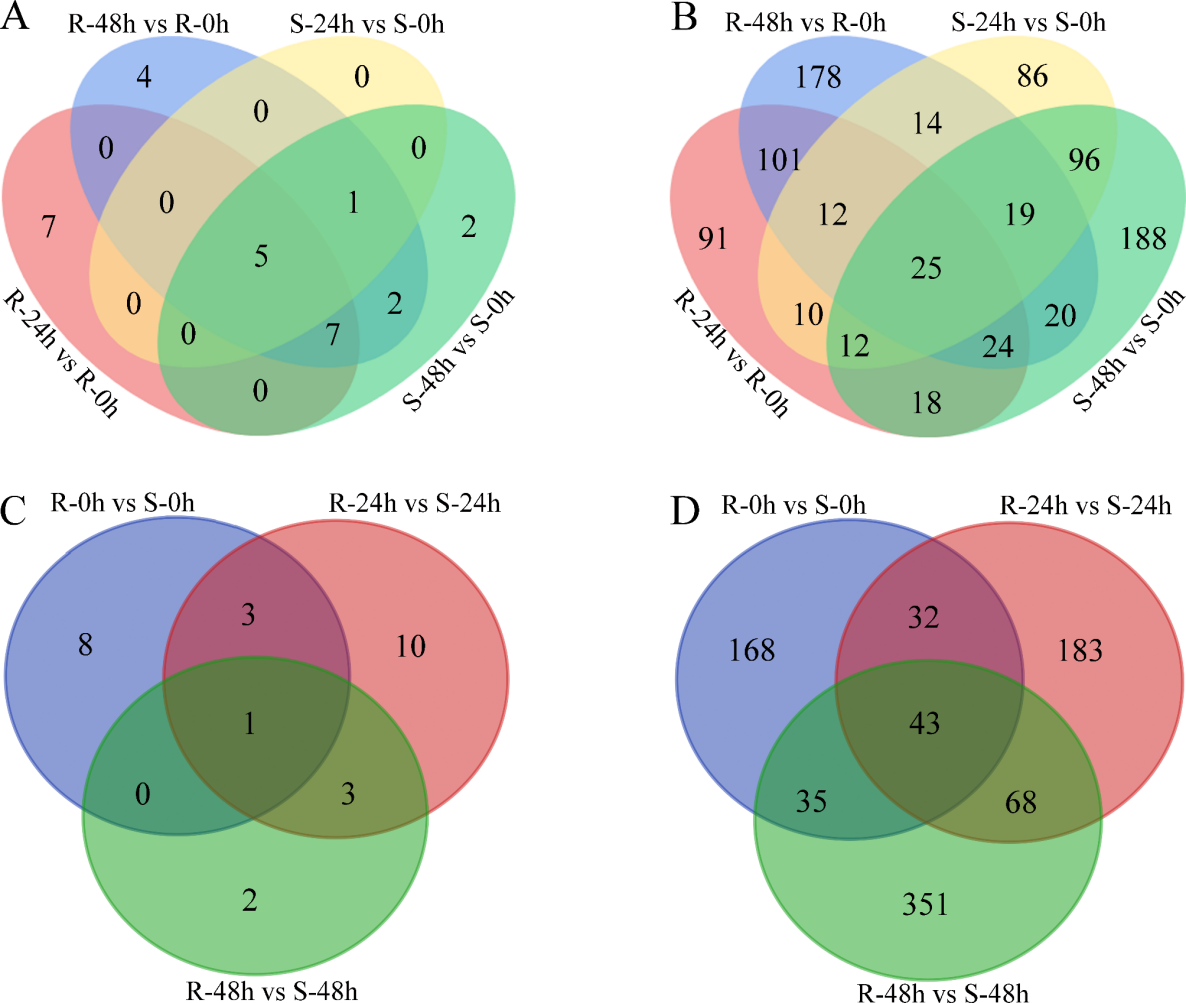
Venn diagrams of known DEMs (**A**) and novel DEMs (**B**) in R and S accessions after *S. sclerotiorum* infection, Venn diagrams of known DEMs (**C**) and novel DEMs (**D**) between R and S accessions after *S. sclerotiorum* infection at the same time point

For screening of miRNA responses to *S. sclerotiorum* infection, 28 known (belonging to 11 families) and 894 novel miRNAs were detected as differentially expressed miRNAs (DEMs) in response to *S. sclerotiorum* infection (Table S6). Among the 28 known DEMs, miR166f was downregulated at 48 h after *S. sclerotiorum* infection in both R and S accessions, while the rest of them were upregulated at 24 h or (and) 48 h after *S. sclerotiorum* infection in R or (and) S genotypes. miR156b/c and miR164b/c/d were identified as DEMs at 24 and 48 h after *S. sclerotiorum* infection in both genotypes. miR156g was a DEM in R-48 h vs. R-0 h, S-24 h vs. S-0 h, and S-48 h vs. S-0 h comparisons; miR156a/d/e/f and miR390a/b/c were DEMs in the R-24 h vs. R-0 h, R-48 h vs. R-0 h, and S-48 h vs. S-0 h comparisons; and miR168a was a DEM in R-48 h vs. R-0 h and S-48 h vs. S-0 h. A set of miRNAs were specifically identified as DEMs in only one comparison, including miR395a/b/c, miR6031, miR171a/b/c/d/e, miR164a, miR172a, miR393, and miR860 (Fig. 3 A-B). Among the 894 novel DEMs, 25 were detected in all comparisons of R-24 h vs. R-0 h, R-48 h vs. R-0 h, S-24 h vs. S-0 h, and S-48 h vs. S-0 h; 91, 178, 86 and 188 DEMs were detected in comparisons of R-24 h vs. R-0 h, R-48 h vs. R-0 h, S-24 h vs. S-0 h, and S-48 h vs. S-0 h, respectively (Fig. 3B).

**Fig. 4:**
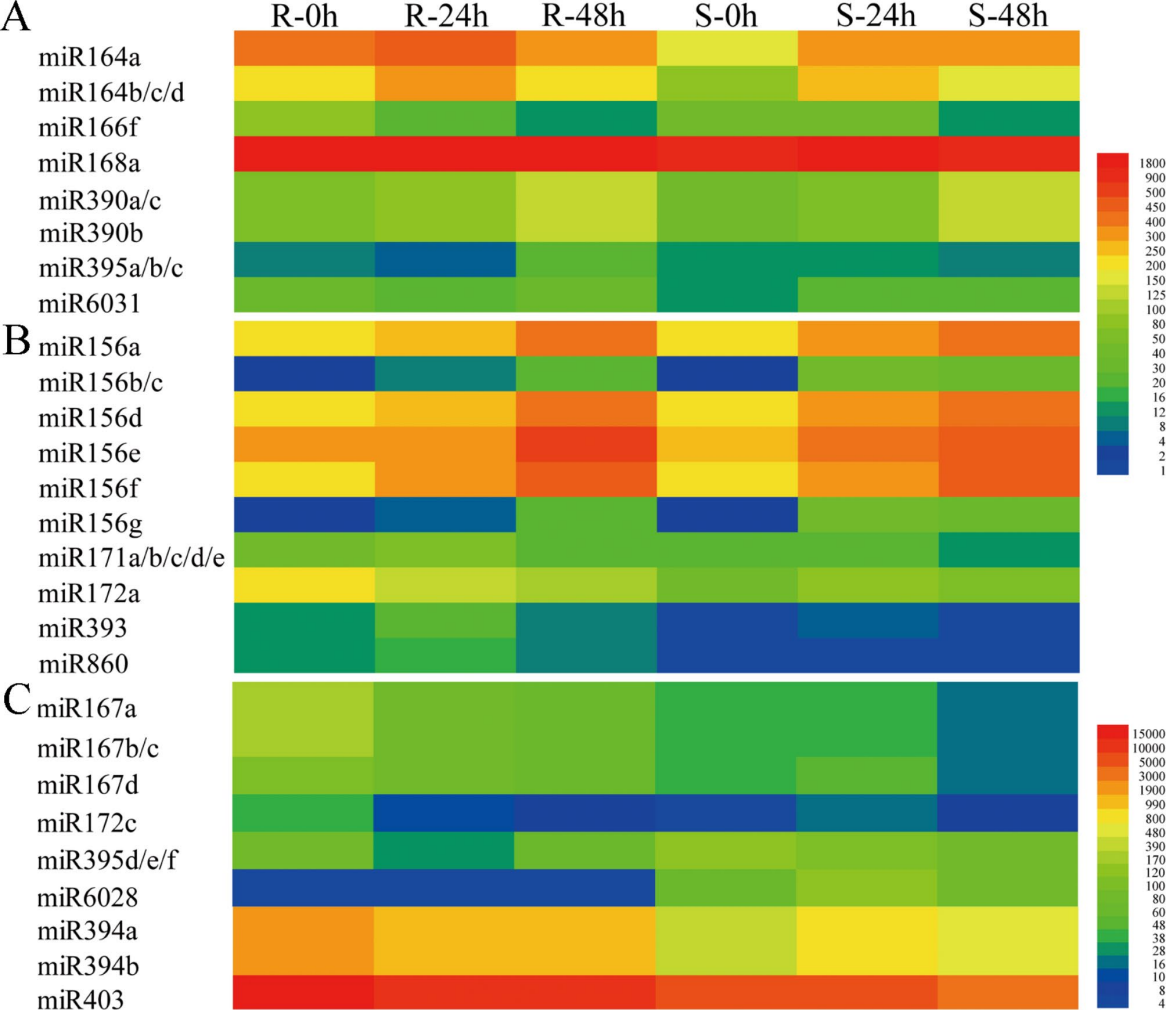
Heatmaps of specific known DEMs (**A**) and common known DEMs (**B**) in R and S accessions after *S. sclerotiorum* infection. Heatmaps of common known DEMs (**B**) and specific known DEMs (**C**) between R and S accessions after *S. sclerotiorum* infection at the same time point

In a comparison of the responsiveness of *S. sclerotiorum* infection between the two genotypes, 27 known DEMs (belonging to 10 families) and 880 novel DEMs were identified (Table S7). Among the 27 known DEMs, miR6028 was downregulated in R accessions compared with S accessions at three time points. miR395d, miR395e, and miR395f were also downregulated in comparisons of R-0 h vs. S-0 h and R-24 h vs. S-24 h; miR167a, miR167b, and miR167c were upregulated in comparisons of R-24 h vs. S-24 h and R-48 h vs. S-48 h; 8, 10, and 2 DEMs were detected in comparisons of R-0 h vs. S-0 h, R-24 h vs. S-24 h, and R-48 h vs. S-48 h, respectively (Figs. 3C and 4B-C). Among the novel DEMs, 43 were commonly detected in all three comparisons, while 168, 183, and 351 DEMs were detected in comparisons of R-0 h vs. S-0 h, R-24 h vs. S-24 h, and R-48 h vs. S-48 h, respectively (Fig. 3D).

### Target prediction and functional analysis of DEMs

miRNAs mainly work through specific cleavage of the target mRNA and negatively regulate target gene expression at the post-transcriptional level. Therefore, to explore the functions of DEMs, we predicted target genes of these DEMs using bioinformatics analysis. In this study, 8,523 target genes, including 206 for 13 known miRNAs and 8393 targets for 569 novel miRNAs, were predicted. To further explore the functions of the *B. napus* miRNAs in response to *S. sclerotiorum* infection, we used Gene Ontology (GO) analysis to assess the potential functions of all annotated targets based on three main categories: biological process, cellular component, and molecular function. We found 16, 15, and 20 functional groups in cellular components (CC), molecular functions (MF), and biological processes (BP), respectively (Fig. S3). Cellular processes (GO: 0009987), cell (GO: 0005623), and binding (GO: 0005488) were the dominant functions in each of the three main categories (Fig. S3).

**Fig. 5:**
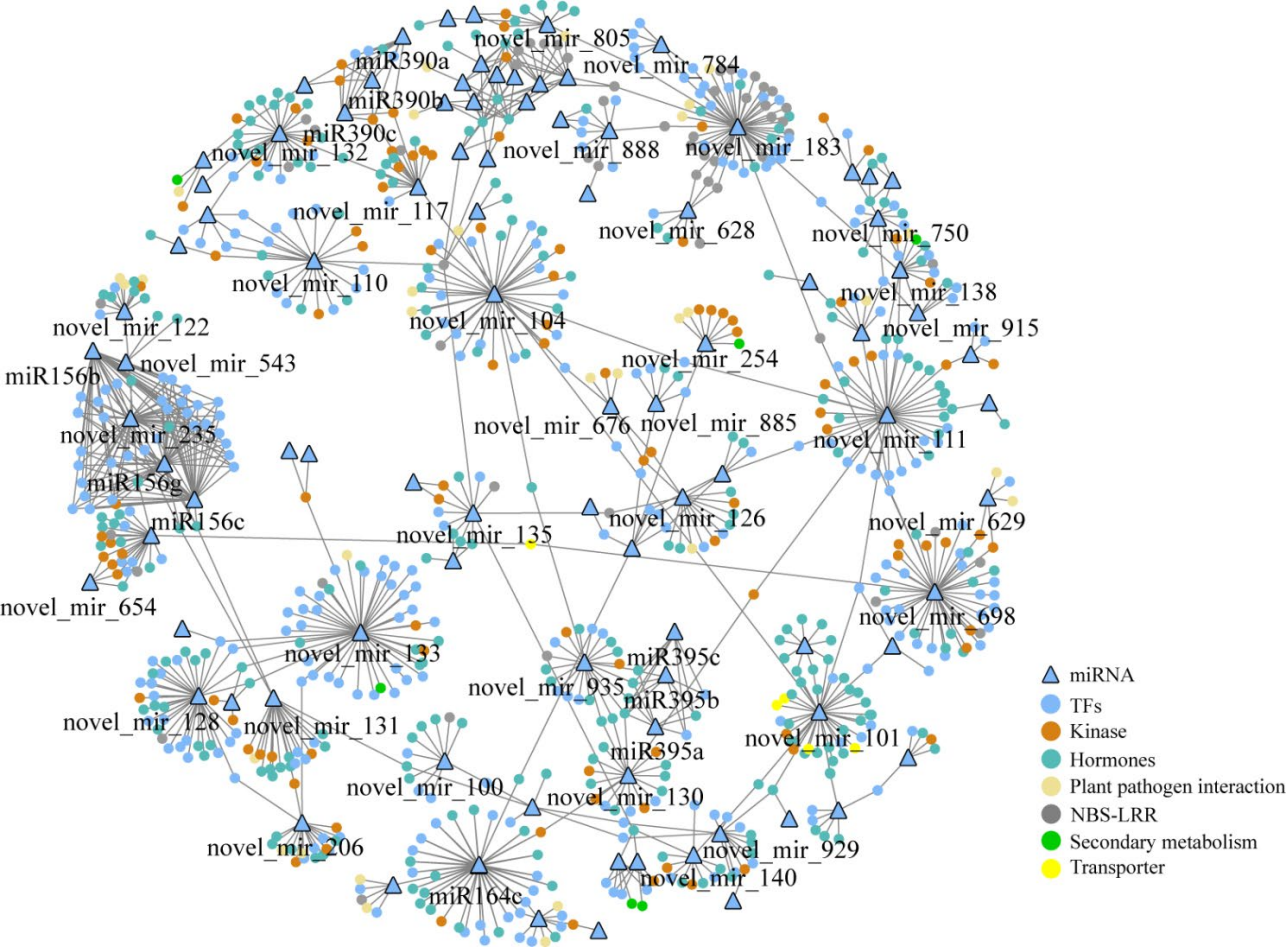
Regulatory network of miRNA-target module response to *S. sclerotiorum* infection in oilseed rape. Solid triangle means miRNAs; solid circle means proteins encoded by target genes; blue circle means TFs; deep yellow circle means kinases; cyan circle means hormones; light yellow circle means plant pathogen interaction proteins; grey circle means NBS-LRR type proteins; green circle means proteins involved in secondary metabolism; yellow circle means transporter proteins

For further analysis of the interaction of small RNAs with target genes in response to *S. sclerotiorum* infection, all target genes were classified and annotated (Table S8). The target genes of small RNAs encoding disease resistance proteins (R proteins), MAPK kinases, transcription factors, and hormone-related proteins were displayed using Cytoscape software (Fig. 5). The results showed that there were 181 genes encoding R protein targeted by 114 DEMs (9 known and 105 novel). The expression of miR156b/c/g was significantly upregulated at both 24 and 48 h in the R and S materials, and its target gene was *BnaA10g09930D*, homologous to *AT5G55830* in *A. thaliana*. The gene encoded the receptor kinase of L-TYPE LECTIN RECEPTOR KINASE S.7, which plays critical roles in disease resistance.

In addition, 22 MAPK cascade pathway genes were targeted by 14 novel DEMs, which were downregulated except novel_mir_443 (Fig. 5 and Table S8). The target genes of novel_mir_443, *BnaA03g38420D* and *BnaC03g45260D*, encoding mitogen-activated protein kinase 3 (MAPKKK3), activate MPK3/6 downstream of multiple pattern recognition receptors and confer resistance to both bacterial and fungal pathogens.

Furthermore, 556 genes belonging to hormone-related genes were targeted by 205 DEMs, and these genes were mostly involved in auxin and abscisic acid metabolism and signal transduction pathways (Fig. S4 and Table S8). Target genes of 13 known DEMs belonging to four miRNA families were involved in hormone metabolism and signal transduction pathways. The target genes of miR156b/c/g encoding 12-oxophytodienoate reductase 1 (OPR1) and OPR3 are necessary for jasmonate biosynthesis. CUP-SHAPED COTYLEDON1 (CUC1) and CUC3 targeted by miR164b/c/d were involved in the auxin pathway, ETHYLENE-INSENSITIVE3-like 3 (EIL3) regulated by miR166f was involved in the ethylene metabolism pathway, and the miR395a target gene, allene oxide cyclase 2, was involved in the jasmonate biosynthesis pathway. Nine-cis-epoxycarotenoid dioxygenase 3 (NCED3) and NCED9, the key members of abscisic acid synthesis and metabolism, were encoded by target genes of novel_mir_120 and novel_mir_736, respectively, and novel_mir_120 was downregulated at both 24 and 48 h in R lines. Novel_mir_736 was only upregulated in the R-48 h sample. Other key hormone genes, such as *SUPPRESSOR OF AUXIN RESISTANCE 3*, *auxin response factor 16* (*ARF16*), *gibberellin 2-oxidase 3*, *jasmonate-zim-domain protein 10*, *ethylene responsive element binding factor 3*, and *serine/threonine protein kinase 1*, were also detected (Fig. S4 and Table S8).

Five hundred and sixty-nine genes targeted by 173 DEMs were belonged transcription factor (TF) genes, of which *MYB*, *NAC*, *bHLH*, *WRKY*, *GRF*, *SBP*, and *HD-ZIP* were the major TF families (Fig. S5 and Table S8). Among them, *SBP* and *NAC* TF genes were mainly regulated by miR156 and miR164, respectively. *GRF* TF genes were regulated by miR390, while *MYB* and *WRKY* were mainly regulated by novel miRNAs (Fig. S5 and Table S8).

### miR156 positively regulates resistance to *S. sclerotiorum* in Arabidopsis

**Fig. 6:**
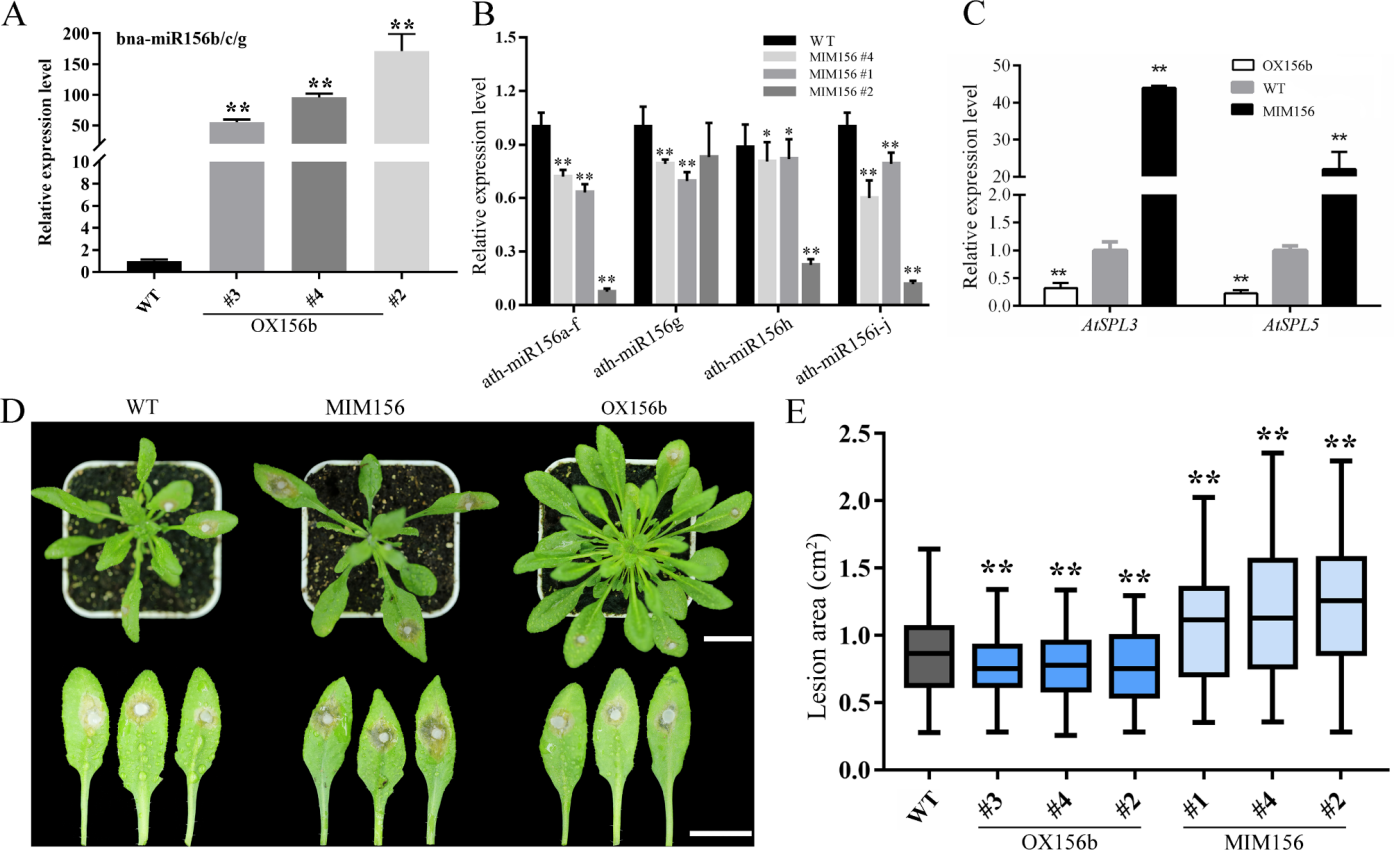
Transformation of Arabidopsis with miR156b and target mimic of miR156 and the disease symptoms of transgenic plants inoculated with *S. sclerotiorum*. (**A**-**B**) qRT-PCR detection of bna-miR156b/c/g and ath-miR156 expression in WT (Col-0), MIM156, and OX156b leaves from 4-week-old Arabidopsis plants. Values are the means ± SDs from three replicates. (**C**) Expression levels of the miR156 target genes *AtSPL3* and *AtSPL5* in WT, MIM156, and OX156b leaves from 4-week-old Arabidopsis plants. Values are the means ± SDs from three replicates. (**D**-**E**) Disease symptoms (**D**) and lesion area (**E**) measurements in WT, OX156b, and MIM156 T_4_ transgenic lines 24 h after *S. sclerotiorum* infection. Bars = 2.0 cm. Data are the means ± SDs from three independent experiments. The significant differences from WT are indicated (Student’s *t* test: **. *P* < 0.01)

The miRNAome sequencing data showed that multiple members of the miR156 family respond to *S. sclerotiorum* infection. Among them, miR156b was significantly upregulated after *S. sclerotiorum* inoculation in both groups at 24 and 48 h after inoculation. Accordingly, we hypothesized that miR156 is involved in oilseed rape resistance to *S. sclerotiorum*. To test this hypothesis, we conducted the following experiments. The conservation analysis of the miR156 precursor sequence and mature sequence in several species shows that miR156 is highly conserved (Fig. S6 A-C). The overexpression of miR156b and target mimic of miR156 in Arabidopsis was performed to determine whether miR156b is required for basal resistance against *S. sclerotiorum* infection. Homozygous overexpression lines of OX156b (#3, #4 and #2) and the MIM156 miR156 target mimic lines MIM156 (#4, #1 and #2) were selected from the positive transgenic Arabidopsis plants to evaluate their resistance to *S. sclerotiorum*. qRT-PCR analysis indicated that miR156b was overexpressed in different OX156b lines, and the expression of each member of miR156 in different MIM156 lines dropped to varying degrees (Fig. 6A and B). We also detected the expression levels of the miR156 target genes *AtSPL3*, *AtSPL5*, *AtSPL6*, *AtSPL10*, *AtSPL11,* and *AtSPL13* in the OX156b and MIM156 lines (Fig. 6C and Fig. S7). Compared with those of the WT, their expression levels were markedly inhibited in OX156b plants; in contrast, they were significantly increased in MIM156 plants. Subsequently, leaf inoculation of Arabidopsis seedlings was used to assess resistance to *S. sclerotiorum* at four weeks of age. The results of three independent *S. sclerotiorum* inoculation experiments showed that disease symptoms were observed at 24 h after inoculation in all plants (Fig. 6D). The phenotype analysis demonstrated that OX156b plants showed much smaller chlorotic/necrotic lesions relative to the WT plants, while MIM156 plants showed a significantly enhanced severity and produced more severe disease symptoms with greater necrotic lesion area than in the WT and OX156b plants after inoculation (Fig. 6D-E).

**Fig. 7:**
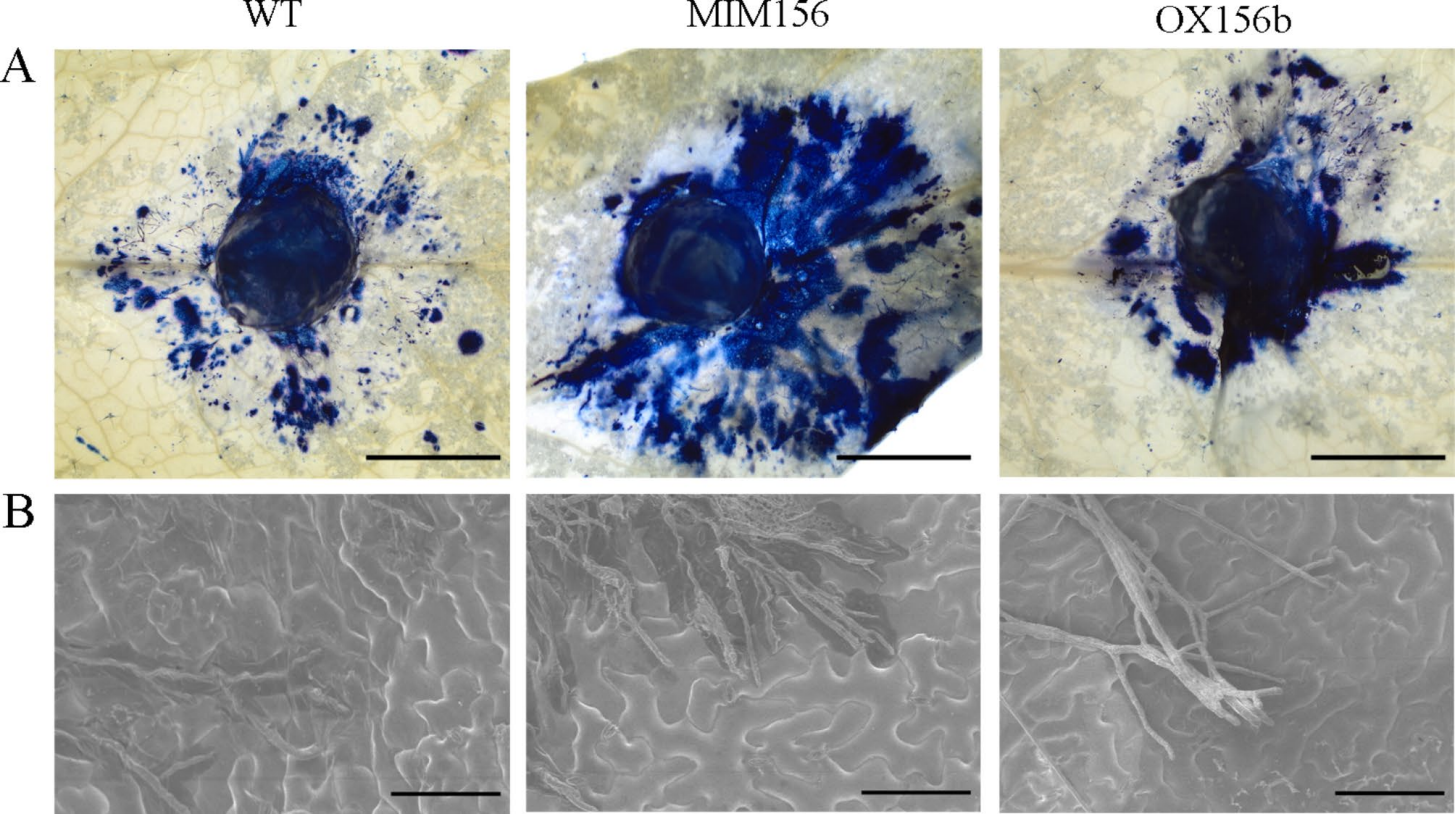
The growth of *S. sclerotiorum* was examined by trypan blue staining and SEM. (**A**) Trypan blue staining of infected leaves of WT (Col-0), MIM156, and OX156b plants at 24 h after inoculation with *S. sclerotiorum*. Bars = 3 mm. (**B**) Representative SEM graphs of *S. sclerotiorum* growth in the leaves of WT (Col-0), MIM156, and OX156b plants at 24 h after inoculation with *S. sclerotiorum.* Bars = 100 μm

Furthermore, a trypan blue staining assay was performed to examine host cell death in WT, OX156b, and MIM156. Among the three, MIM156 had the most extensive and rapid cell death of necrotic lesions following inoculation with *S. sclerotiorum* (Fig. 7A). Mycelial growth was aggregated in the necrotic zone in OX156b leaves with a smaller staining area than in WT and MIM156 leaves (Fig. 7A). The staining results were consistent with the phenotype analysis of *S. sclerotiorum* inoculation. The hyphal growth of *S. sclerotiorum* in the infected leaf tissues was also examined by scanning electron microscopy (SEM). The SEM results showed that a large number of mycelia of wild type 1980 strain gathered together on the surface of MIM156 leaves to form an infection cushion and infected the leaves (Fig. 7B). Necrotic cells were produced in all places touched by the mycelia, which had more serious symptoms than those of the WT (Fig. 7B). In contrast, the mycelia crawled on the leaf surface of OX156b to form infection cushion, but no obvious necrotic cells were produced, and there were fewer symptoms than those of the WT (Fig. 7B). These results suggest that miR156b is a critical regulatory factor in *S. sclerotiorum* resistance and that the overexpression of miR156b in Arabidopsis increased resistance to *S. sclerotiorum* infection. Conversely, downregulation of miR156b enhanced susceptibility to *S. sclerotiorum*.

### qRT-PCR validation of miRNAs and corresponding target genes

**Fig. 8:**
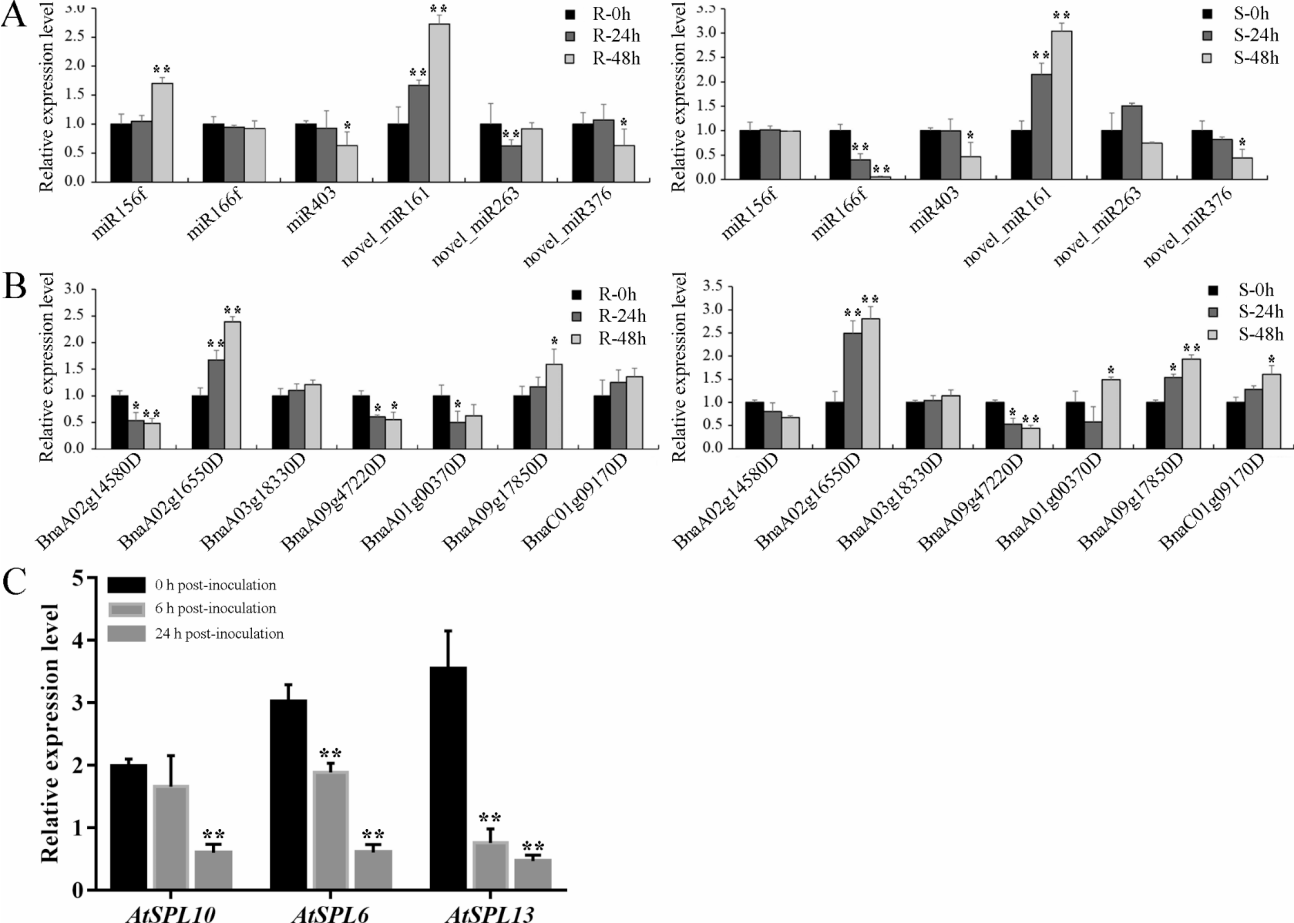
Relative expression levels of miRNAs and their corresponding targets. (**A-B**) The expression profiles of DEMs and their corresponding targets in the six libraries at 0 hpi, 24 hpi, and 48 hpi after *S. sclerotiorum* infection. Values are the mean ± SD of three biological replicates. The significant differences from R-0 h and S-0 h are indicated, respectively (Student’s *t* test: **. *P* < 0.01). (**C**) Relative expression levels of *AtSPL10*, *AtSPL6*, and *AtSPL13* in leaves of 4-week-old WT plants inoculated with *S. sclerotiorum* at 0 hpi, 6 hpi, and 24 hpi. Values are the mean ± SD of three biological replicates. The significant differences from WT are indicated (Student’s *t* test: **. *P* < 0.01)

To confirm the expression patterns of the miRNAs in response to *S. sclerotiorum* infection, we performed quantitative real-time polymerase chain reaction (qRT-PCR) for three known miRNAs (miR403, miR156f, and miR166f) and three novel miRNAs (novel_mir_161, novel_mir_263, and novel_mir_376) (Fig. 8A). As expected, the qRT-PCR results showed a high degree of similarity with the expression profiles obtained by RNA-seq. For the known miRNAs, transcripts of miR156f and novel_mir_161 were upregulated and transcripts of miR166f and miR403 were downregulated, remaining at an extremely low expression level, at the three time points in both the R and S libraries. We also performed qRT-PCR on the target genes (*BnaA02g14580D*, *BnaA02g16550D*, *BnaA03g18330D*, *BnaA09g47220D*, *BnaA01g00370D*, *BnaA09g17850D,* and *BnaC01g09170D*) of six *S. sclerotiorum* stress-responsive miRNAs (Fig. 8B). The expression of the seven target genes showed an inverse relationship with the expression of their corresponding miRNAs, which was expected and further confirmed the accuracy of the sequencing results.

The expression levels of *AtSPL6*, *AtSPL10*, and *AtSPL13* were detected at 0 h, 6 h, and 24 h in WT plants after inoculation with *S. sclerotiorum*. The expression of all these genes was significantly downregulated after inoculation compared with before inoculation, and *AtSPL13* was the most downregulated (Fig. 8C). These results indicate that *SPLs* may participate in the negative regulation of resistance to *S. sclerotiorum* in Arabidopsis. We analyzed 56 predictive target genes of 34 miRNAs using transcriptome data of resistant *B. napus* obtained in our laboratory before and after inoculation with *S. sclerotiorum* (0 and 48 h) [17]. The data shows that multiple target genes of DEMs, including miR156, miR164, miR395, miR390, and many novel miRNAs, are differentially expressed after inoculation with *S. sclerotiorum* (Fig. S8). The most significant ones are *SPL2*, *SPL9*, *SPL10*, *SPL11*, *SPL13*, *OPR3*, *AOC2*, *LOX2*, *NAC080*, and *DOX1*, indicating that these genes may be targeted and regulated by miRNAs to participate in resistance to *S. sclerotiorum* in rapeseed.

## Discussion

### Differential expression of miRNA induced by *S. sclerotiorum* infection in rapeseed

A set of DEMs was identified by using high throughput sequencing with genome wide identification and functional prediction, which respond to *S. sclerotiorum* infection in *B. napus*, including miR156, miR166, miR6028, miR824, miR169, miR394, miR390, miR395, and miR396 [16, 17, 24, 25]. In our study, a total of 40 known and 1105 novel miRNAs were detected as DEMs in response to *S. sclerotiorum* infection, including miR156a/b/c/d/e/f/g, miR6028, miR394a/b, miR390a/b/c, miR395a/b/c and miR166f (Fig. 4). Except for miR166f, other DEMs were upregulated to resist *S. sclerotiorum* infection on the stem in comparisons of R-24 h vs. R-0 h, R-48 h vs. R-0 h, S-24 h vs. S-0 h, and S-48 h vs. S-0 h, and miR156 was the most prominent (Fig. 4 and Table S6). miR156 has been previously reported to be significantly upregulated after infection with fungal phytopathogens such as *D. gregaria* and *B. cinereal* [14, 15]. Multiple miR156 members screened as upregulated DEMs in response to *Sclerotinia* stem rot (SSR) in oilseed rape [16, 17]. In addition, miR171a/b/c/d/e, miR167a/b/c/d, miR164a/b/c/d, and 172a/c were identified as DEMs in this study (Fig. 4). Previous research shows that overexpression of miR171b increased rice blast resistance accompanied by enhanced defence responses, whereas blocking miR171b expression in rice led to greater susceptibility to blast disease, associated with compromised defence responses [26]. The Zma-miR167-*ZmARF3*/*30* module restricts maize chlorotic mottle virus (MCMV) infection by regulating *polyamine oxidase 1* expression, while MCMV encodes the p31 protein to counteract this defence response [27]. The differential expression of these miRNAs positively modulates the degree of resistance to *S. sclerotiorum* in oilseed, and these crucial miRNAs regulate host plant defence mechanisms by directly targeting defence-related genes.

### *R* genes involved in response to *S. sclerotiorum* infection

*R* genes, namely, receptors with nucleotide-binding domains and leucine-rich repeats (NLRs), can detect effectors to help pathogens infect hosts [28]. In rice, the CNL protein RGA5 directly binds to the *M. oryzae* effectors *Avr*-Pia and Avr1-Co39 [29]. More research is still necessary to elucidate the functions of NLRs, such as stem rot resistance genes, in response to pathogen attack. In this study, 181 genes were predicted as target genes for 114 (9 known and 105 novel) miRNAs. One target gene, *BnaA10g09930D* for miR156b/c/g, two targets, *BnaAnng09060D* and *BnaC06g36320D* for miR164b/c/d, and four copies of the receptor-like protein 46 gene for miR390a/b/c were detected.

### Target genes involved in signal transduction in response to *S. sclerotiorum* infection

Mitogen-activated protein kinase cascades have been reported to be involved in the response to (a)biotic stresses [30, 31]. MPK8 connects protein phosphorylation, Ca^2+^, and ROS in the signalling pathway [32]. In our study, four target genes of three novel miRNAs encoding two MPK8s, one MPK9 and one MPK17 family gene were detected. MKK6, interacting with MAPKKK5, which is a potential substrate of the receptor-like cytoplasmic kinase BRASSINOSTEROID-SIGNALINGKINASE1 plays key roles in the MAPK cascade response to the immune pathway [33]. Two novel miRNAs, novel_mir_110 and novel_mir_698, targeting two copies of MKK6 genes were also detected in this study. MAPKKK5 is involved in the regulation of the defence response to fungi [33]. Eleven target genes of seven novel miRNAs encoding two MAPKKK3s, three MAPKKK5s, one MAPKKK13, one MAPKKK14, three MAPKKK15s and one MAPKKK16 were detected. Calcium-dependent protein kinase family genes play crucial roles in the immune response pathway [34]. Two novel miRNAs, novel_mir_177 and novel_mir_121, target five genes encoding two copies of calcium-dependent protein kinase 10 (CPK10) and three CPK28s.

### Target genes involved in hormone regulation in response to *S. sclerotiorum* infection

Hormones play critical roles in adapting to adverse environmental conditions, such as abiotic stresses (salt, drought, and extreme temperatures) and biotic stresses (bacteria, fungi, and viruses) [35]. In this study, 556 target genes of 205 (13 known and 192 novel) miRNAs were involved in the biosynthesis or signal transduction of hormones. Among them, 48 target genes of 32 miRNAs encode proteins are involved in jasmonic acid (JA) biosynthesis, including 12-oxophytodienoate reductase 1 (targeted by miR156b/c/g and novel_mir_131), allene oxide cyclase 2 (targeted by miR395b/c/g, novel_mir_130 and novel_mir_935), fatty acid desaturase 3 (targeted by novel_mir_132), and lipoxygenase 2 family proteins (targeted by novel_mir_227 and novel_mir_614). Three genes encoding jasmonate-zim-domain protein 5, which negatively regulates JA transcriptional activity, were targeted by novel_mir_128 and novel_mir_477. miR164b/c/d targeted NAC domain containing protein 80 (NAC80) and NAC100 are involved in brassinosteroid signalling.

### Target genes encoding TFs playing an essential role in the response to *S. sclerotiorum* infection

TFs play regulatory roles in response to pathogen attack in several aspects [36, 37]. TF families, such as NAC and WRKY, are especially involved in plant defence processes [38, 39]. In barley, *HvNAC6* was upregulated after *Blumeria graminis* infection [40]. WRKY TFs not only participate in abiotic stresses but are also involved in the response to pathogen attack [41]. In this study, 569 genes encoding 43 TF families were predicted as target genes for 173 (13 known and 160 novel) miRNAs. Among them, 46 target genes for miR164b/c/d and 20 novel miRNAs belonged to NAC TFs. Thirty-two target genes for 11 novel miRNAs encode WRKY TFs.

### miR156 regulated *S. sclerotiorum* resistance

In this study, OX156b enhanced the resistance of *S. sclerotiorum* in transgenic Arabidopsis, resulting in mild disease symptoms in subsequent inoculation experiments (Fig. 6). In Arabidopsis, miR156-targeted SPL/SBP box transcription factors SPLs positively and directly regulate the MADS box genes *APETALA1* and *FRUITFULL* and the central regulator of flowering *LEAFY3* and *FLOWERING LOCUS T* to control the timing of flower formation and fruit development [42, 43]. Much larger chlorotic/necrotic lesions were observed in MIM156 plants than in WT plants and showed stronger necrosed tissues around the infection cushion (Fig. 6). In addition, the downregulation of *AtSPL10*, *AtSPL6*, and *AtSPL13* expression following *S. sclerotiorum* treatment was detected in WT (Fig. 7). *SPL2*, *SPL9*, *SPL10*, *SPL11*, and *SPL13* were significantly downregulated in resistant *B. napus* after inoculation with *S. sclerotiorum* (Fig. S7). These results suggested that SPLs might act as target genes involved in miR156-modulated SSR defence against the necrotrophic fungus *S. sclerotiorum*. Previous studies have also demonstrated that the miR156/SPL module positively regulates plant biotic and abiotic stress responses [19, 44]. miR156 is a positive regulatory factor that resists *B. cinerea*, and the miR156 target gene *SPL9* negatively affects the response of Arabidopsis to this necrotrophic pathogen [18]. Previous research has also suggested that miR156 negatively regulates resistance to biotic stress. miR156 negatively regulates the resistance of the hemibiotrophic necrotrophic pathogen *Xoo*, and the miR156 target gene *OsSPL7* enhances disease resistance to bacterial blight [20]. The repression of miR156 and overexpression of *SPL9* enhanced resistance to *Pst* DC3000 infection in Arabidopsis [21]. Moreover, miR156fhl-3p and miR156h negatively regulate the disease resistance of rice blast in rice, but miR156fhl-3p was differentially responsive to *M. oryzae* in susceptible and resistant accessions [23]. The reason for this difference may be that miR156 plays different roles in different species under different abiotic stresses and biotic stresses with distinct underlying molecular mechanisms. The miR156/SPLs module can not only strengthen plant resistance but also weaken plant resistance to various abiotic and biotic stresses, which may involve extremely complex regulatory networks.

## Conclusions

Heavy losses in oilseed rape are caused by *Sclerotinia* stem rot every year. Thus, exploring the molecular mechanisms of resistance to the *S. sclerotiorum* pathogen is a prerequisite for breeding resistant rapeseed varieties. In current study, DEMs in *B. napus* stems after *S. sclerotiorum* inoculation were identified. There was a dramatic difference between the two types (R and S) of oilseed rape in miRNA transcriptome response to *S. sclerotiorum* infection. The target genes of these DEMs may be involved in regulating resistance to *S. sclerotiorum* in rapeseed by encoding R proteins, signal transduction proteins, hormones, and transcription factors. Overexpressing miR156b markedly strengthened Arabidopsis resistance against *S. sclerotiorum*. Therefore, miR156 positively regulates rapeseed resistance to *S. sclerotiorum*. This study provides theoretical bases for the genetic improvement of rapeseed resistance to *S. sclerotiorum*.

## Materials and methods

### Plant materials and growth conditions

Five resistant (R, namely, WH-57, 2011–7103, Huayou14, Chuxianbaihua, and Youyan2) and five susceptible (S, namely, Yangjian8, P685, Guangde138, SWU69, and 07037) *B. napus* winter-type accessions were identified in our previous study and were used in miRNA-seq experiments [45]. The seeds of the rapeseed cultivars mentioned above were grown in the experimental farm with conventional management. Arabidopsis wild-type plants (ecotype Columbia, Col-0) and transgenic lines were sown in flower pots (1 seedling/pot) and grown in a growth chamber set at 22 ℃ with 80 ± 5% relative humidity and a 16 h light (150 mmol m^-2^ s^-1^)/8 h dark photoperiod. *S. sclerotiorum* wild type 1980 strain was provided by Dr. Jiaqin Mei from Southwest University and was subcultured on potato dextrose agar medium prior to inoculation (20% potato, 2% dextrose, and 1.5% agar) at 22 °C in darkness.

### Plasmid construction and genetic transformation of Arabidopsis

The artificial target mimic of miR156 was acquired by annealing with primers MIM156F and MIM156R with gene splicing by overlap extension PCR (Table S2). The target mimic of miR156 fragments were inserted into *INDUCED BY PHOSPHATE STARVATION1* to replace the miR399 target site [46]. Then, the DNA fragments were cloned into the vector PEaryGate101, resulting in the overexpression construct MIM156. To obtain transgenic plants overexpressing miR156b, we amplified the precursor sequence of miR156b from the rapeseed cultivar ‘ZS11’ genomic DNA from the rapeseed cultivar ‘ZS11’ cDNA with primer combinations (Table S2). Subsequently, the amplified and purified fragments were cloned into expression vector pEarleyGate101, resulting in the overexpression construct OX156b. Then, the recombinant plasmids were inserted into the Arabidopsis genome via the pollen tube pathway method mediated by *Agrobacterium* strain GV3101 [47]. After positive transformants were screened and self-crossed, the seeds of Arabidopsis T_4_ transgenic homozygous and WT lines were sown and used in follow-up experiments.

### Pathogen inoculation, disease assay, phenotype analysis, and tissue harvest

The R and S *B. napus* plants were selected for inoculation and each randomly selected sample had three biological replicates, where each replicate consisted of 30 plants for three time points (0, 24, and 48 hpi). Stems of these *B. napus* accessions were inoculated according to the procedure described previously [45]. Infectious stem tissues harvested from each treatment at each time point (five individual plants with 30 inoculation sites) were pooled as one sample for miRNA-seq and qRT-PCR. The tissues harvested above were frozen immediately in liquid nitrogen and stored at -80 °C for RNA extraction. The Arabidopsis seedlings of WT and two transgenic lines (MIM156 and OX156b) were subjected to leaf inoculation tests to assess the resistance to *S. sclerotiorum* at four weeks of age according to the reported method [48], and each sample had three independent experimental replications, where each replicate consisted of 20 plants and no less than three leaves of each plant were inoculated. The data were analyzed by two-tailed Student’s *t* test with significant differences (*P* < 0.05). The infected leaves were harvested at 24 h following inoculation for trypan blue staining and scanning electron microscopy (SEM). Fresh rosette leaves from WT and two transgenic lines at four weeks of age were sampled for qRT-PCR with three biological replicates.

### Total RNA extraction, sRNA library construction and deep sequencing

Total RNA of all six samples was extracted according to our previous studies [17]. The quantity of the total RNA was detected using a Bioanalyzer 2100 and evaluated by electrophoresis on a 1% agarose gel. Six sRNA libraries (S-0 h, S-24 h, S-48 h, R-0 h, R-24 h, and R-48 h) were constructed and deep sequenced using an Illumina HiSeqTM2000 at the Beijing Genomics Institute (BGI; Shenzhen, China).

### Identification of known and novel miRNAs in *B. napus*

Clean reads were obtained after removing the adapter dimers, junk, low complexity reads of all raw data. Then, the clean reads of each sample were used to search GenBank and the Rfam database (https://www.sanger.ac.uk/science/tools) to annotate rRNAs, tRNAs, snRNAs, and snoRNAs. Reads belonging to repeat sequences, exons and introns in the *B. napus* genome (http://www.genoscope.cns.fr/brassicanapus/) [49] and reads matching miRNAs in the miRBase v.22.0 database (http://www.mirbase.org/) [50] were also annotated. Known miRNAs in *B. napus* were identified with up to one mismatch against the *B. napus* species in the miRBase v.22.0 database. The remaining sequences were then analysed to predict novel miRNAs according to our previous studies [17].

### Differential expression analysis of miRNAs after *S. sclerotiorum* infection

For screening of miRNA responses to *S. sclerotiorum* infection, their expression, including those of known and novel miRNAs in each sample, was normalized using the following formula: normalized expression = actual miRNA count/total count of clean reads × 1,000,0. The expression value was regarded as 0.01 for further analysis if the read count of a miRNA was 0. The miRNAs were regarded as DEMs if they met the following criteria: ∣log_2_(FCs)∣ ≥1 and *q*-value ≤ 0.05.

### Target gene prediction of DEMs and functional analysis

For further analysis of the functions of DEMs, the website psRNATarget 2011 (http://plantgrn.noble.org/psRNATarget/) [51] was used to predict target genes of miRNAs according to default parameters. Furthermore, Blast2GO 5.0 software (https://www.blast2go.com/) was used to analyse the functional annotation of all target genes. Target genes involved in different processes were annotated and graphical visualization of the miRNA-gene networks was performed using Cytoscape 3.0 (http://www.cytoscape.org/).

### Trypan blue staining and scanning electron microscopy

Cell death in the leaves of transgenic plants and WT plants at 24 h post-inoculation was detected using trypan blue staining according to the procedures described previously [52]. In brief, inoculated leaves were soaked with trypan blue staining solution containing 2.5 mg ml-1 trypan blue and then incubated for 8 h at 25 °C. Subsequently, leaves were transferred to the corresponding destaining solution and incubated at 65 °C for 30 min. The leaves were then incubated in fresh destaining solution at room temperature until completely destained and preserved in 10% glycerol. Images were obtained using the CMOS colour camera OLYMPUS LC30 and zoom stereo microscopes SZ61 (Olympus, Tokyo, Japan) under bright-field conditions. The hyphal growth of *S. sclerotiorum* in the infected leaf tissues of transgenic plants and WT plants at 24 h post-inoculation was examined by SEM. Images were obtained using a SU3500 scanning electron microscope (Hitachi, Tokyo, Japan).

### Validation of the miRNA expression profiles and their targets

qRT-PCR was used to confirm the accuracy of miRNA expression levels and corresponding targets. According to our previous study [17], cDNA for miRNA and target genes was synthesized using the miRcute miRNA FirstStrand cDNA Synthesis Kit (Tiangen, Beijing, China) and iScript cDNA Synthesis Kit (Bio-Rad Laboratories, Inc., Berkeley, California), respectively. qRT-PCR for miRNA and target genes was conducted on a CFX96 Real-time System (Bio-Rad, USA) using a miRcute miRNA qPCR Detection Kit (SYBR Green; Tiangen, Beijing, China) and SsoAdvanced Universal SYBR Green Supermix (Bio-Rad Laboratories, Inc., Berkeley, California), respectively, according to the manufacturer’s instructions. *BnaActin7*, *AtActin2*, and U6 snRNA were used as internal references for miRNAs and corresponding target gene expression levels. The 2^−ΔΔCT^ method was used to calculate the relative expression levels of miRNAs and corresponding targets [53]. The primers used in this study are listed in Table S2, and all samples were subjected to three technical replicates with three biological replicates.

### Electronic supplementary material

Below is the link to the electronic supplementary material.

**Supplementary Material 1: Fig. S1** Length distribution of novel RNAs predicted in this study

**Supplementary Material 2: Fig. S2** First base bias of 21-nt novel miRNAs. The y-axis represents the frequency of nucleotides and the x-axis represents different libraries. Four different colours in the bars represent the four nucleotides

**Supplementary Material 3: Fig. S3** GO annotation of all target genes. The y-axis (left) represents the percentages of genes identified in this study, and the y-axis (right) represents the actual gene number. The genes were annotated in three main categories

**Supplementary Material 4: Fig. S4** Hormone-related genes targeted by miRNAs were shown to be involved in regulatory network mechanisms of miRNA-target module response to *S. sclerotiorum* infection in *B. napus*. Red triangle means miRNAs; solid circle means target genes involved in hormone metabolism and signal transduction; black circle means auxin-related genes; blue circle means abscisic acid-related genes; grey circle means ethylene-related genes; green circle means salicylic acid-related genes; yellow circle means cytokinin-related genes; red circle means brassinosteroid; light blue means gibberellin-related genes; orange yellow means jasmonic acid-related genes

**Supplementary Material 5: Fig. S5** TF genes targeted by miRNAs were found to be involved in regulatory network mechanisms of miRNA-target module response to *S. sclerotiorum* infection in *B. napus*. Red triangle means miRNAs; solid circle means target genes encoding TF genes; blue circle means MYB genes; green circle means NAC genes; light blue means bHLH genes; black circle means GRF genes; purple means HD-ZIP genes; yellow means SBP genes; pink circle means WRKY genes

**Supplementary Material 6: Fig. S6** Multiple sequence alignment result analysis. (A) Multiple sequence alignment of precursor sequences of miR156 family members from Arabidopsis and rapeseed. (B-C) Multiple sequence alignment of mature sequences of miR156 family members from several species. *bna* means *Brassica napus*; *bra* means *Brassica rapa*; *ath* means *Arabidopsis thaliana*; *osa* means *Oryza sativa*; *ptc* means *Populus trichocarpa*; *zma* means *Zea mays*

**Supplementary Material 7: Fig. S7** Expression levels of the miR156 target genes *AtSPL6*, *AtSPL10*, *AtSPL11,* and *AtSPL13* in WT, MIM156, and OX156b leaves from 4-week-old Arabidopsis plants. Values are the means ± SDs from three replicates. Data are the means ± SDs from three independent experiments. The significant differences from WT are indicated (Student’s *t* test: **. *P* < 0.01)

**Supplementary Material 8: Fig. S8** Heatmap of 56 predicted target genes for DEMs in resistant *B. napus* after inoculation with *S. sclerotiorum*

**Supplementary Material 9: Table. S1** Annotation statistics for clean tags generated by the six sRNA libraries; **Table. S2** qRT-PCR primers used in this study; **Table. S3** Summary of small RNA reads obtained in this study; **Table. S4** Information on known miRNAs identified in this study; **Table. S5** Information on novel miRNAs predicted in this study; **Table. S6** Identification of DEMs in R and S accessions after *S. sclerotiorum* infection; **Table. S7** Identification of DEMs between R and S accessions after *S. sclerotiorum* infection at the same time point; **Table. S8** Target prediction of DEMs in R and S accessions after *S. sclerotiorum* infection

## Data Availability

These sequencing data that support the findings of this study were deposited at NCBI with accession number SRP105239.

## Abbreviations

AFB2: Auxin signalling F-box protein 2
*B. cinereal*: *Botrytis cinereal*
*B. napus*: *Brassica napus* L.
CPK10: Calcium-dependent protein kinase 10
CUC1: CUP-SHAPED COTYLEDON1
DEMs: Differentially expressed miRNAs
*D. gregaria*: *Dothiorella gregari*a
GO: Gene Ontology
GRF1/3: Growth regulating factor 1/3
MAPKKK3: Mitogen-activated protein kinase 3
*M. oryzae*: *Magnaporthe oryzae*
MCMV: Maize chlorotic mottle virus
NCED3: Nine-cis-epoxycarotenoid dioxygenase 3
NLRs: Leucine-rich repeats
OPR1: 12-oxophytodienoate reductase 1
*Pst*: *Pseudomonas syringae* pv. Tomato
qRT-PCR: Quantitative real-time polymerase chain reaction
R proteins: Resistance proteins
*S. sclerotiorum*: *Sclerotinia sclerotiorum* (Lib.)
SSR: *Sclerotinia* stem rot
SEM: Scanning electron microscopy
*SPL9*: Squamosa promoter binding protein-like
TIR1: transport inhibitor response 1

## Declarations

### Research involving plants

We confirm that the experimental material is owned by the authors, and deposited in the Chongqing Engineering Research Center for Rapeseed of Southwest University, China.

## References

[1] Bolton MD, Thomma BP, Nelson BD, Nelson (2006). *Sclerotinia sclerotiorum* (Lib.) De Bary: biology and molecular traits of a cosmopolitan pathogen. Molecular Plant Pathology.

[2] Seifbarghi S, Borhan MH, Wei Y, Coutu C, Robinson SJ, Hegedus DD (2017). Changes in the *Sclerotinia sclerotiorum* transcriptome during infection of *Brassica napus*. BMC Genomics.

[3] Tarver JE, Sperling EA, Nailor A, Heimberg AM, Robinson JM, King BL (2013). miRNAs: small genes with big potential in metazoan phylogenetics. Molecular Biology and Evolution.

[4] Achard P, Herr A, Baulcombe DC, Harberd NP (2004). Modulation of floral development by a gibberellin-regulated microRNA. Development.

[5] Guo HS, Xie Q, Fei JF, Chua NH, Chua (2005). MicroRNA directs mRNA cleavage of the transcription factor *NAC1* to downregulate auxin signals for *arabidopsis* lateral root development. The Plant Cell.

[6] Zhou X, Wang G, Sutoh K, Zhu JK, Zhang W (2008). Identification of cold-inducible microRNAs in plants by transcriptome analysis. Biochimica et Biophysica Acta.

[7] Navarro L, Dunoyer P, Jay F, Arnold B, Dharmasiri N, Estelle M (2006). A plant miRNA contributes to antibacterial resistance by repressing auxin signaling. Science.

[8] Sullivan CS, Ganem D (2005). MicroRNAs and viral infection. Molecular Cell.

[9] Hewezi T, Maier TR, Nettleton D, Baum TJ (2012). The *Arabidopsis* MicroRNA396-*GRF1*/*GRF3* Regulatory Module Acts as a Developmental Regulator in the reprogramming of Root cells during Cyst Nematode infection. Plant Physiology.

[10] Robert-Seilaniantz A, MacLean D, Jikumaru Y, Hill L, Yamaguchi S, Kamiya Y (2011). The microRNA miR393 re-directs secondary metabolite biosynthesis away from camalexin and towards glucosinolates. The Plant Journal.

[11] He XF, Fang YY, Feng L, Guo HS (2008). Characterization of conserved and novel microRNAs and their targets, including a TuMV-induced TIR-NBS-LRR class *R* gene-derived novel miRNA in *Brassica*. FEBS Letters.

[12] Li F, Pignatta D, Bendix C, Brunkard JO, Cohn MM, Tung J (2012). MicroRNA regulation of plant innate immune receptors. Proceedings of the National Academy of Sciences of the United States of America.

[13] Li Y, Lu YG, Shi Y, Wu L, Xu YJ, Huang F (2014). Multiple Rice MicroRNAs are involved in immunity against the Blast Fungus *Magnaporthe oryzae*. Plant Physiology.

[14] Chen L, Ren Y, Zhang Y, Xu J, Zhang Z, Wang Y (2012). Genome-wide profiling of novel and conserved *Populus* microRNAs involved in pathogen stress response by deep sequencing. Planta.

[15] Jin W, Wu F (2015). Characterization of miRNAs associated with *Botrytis cinerea* infection of tomato leaves. BMC Plant Biology.

[16] Joshi RK, Megha S, Basu U, Rahman MH, Kav NN, Kav. Genome wide identification and functional prediction of long non-coding RNAs responsive to *Sclerotinia sclerotiorum* infection in *Brassica napus*. Plos One. 2016;11:e0158784. 10.1371/journal.pone.0158784 10.1371/journal.pone.0158784 PMC4936718 27388760

[17] Jian H, Ma J, Wei L, Liu P, Zhang A, Yang B (2018). Integrated mRNA, sRNA, and degradome sequencing reveal oilseed rape complex responses to *Sclerotinia sclerotiorum* (Lib.) Infection. Scientific Reports.

[18] Sun T, Zhou Q, Zhou Z, Song Y, Li Y, Wang HB (2022). SQUINT positively regulates resistance to the Pathogen Botrytis cinerea via miR156-SPL9 Module in Arabidopsis. Plant and Cell Physiology.

[19] Mao YB, Liu YQ, Chen DY, Chen FY, Fang X, Hong GJ (2017). Jasmonate response decay and defense metabolite accumulation contributes to age-regulated dynamics of plant insect resistance. Nature Communications.

[20] Liu M, Shi Z, Zhang X, Wang M, Zhang L, Zheng K (2019). Inducible overexpression of *Ideal Plant Architecture1* improves both yield and disease resistance in rice. Nature Plants.

[21] Yin H, Hong G, Li L, Zhang X, Kong Y, Sun Z (2019). miR156/*SPL9* regulates reactive Oxygen Species Accumulation and Immune Response in *Arabidopsis thaliana*. Phytopathology.

[22] Ge YF, Han JY, Zhou GX, Xu YM, Ding Y, Shi M (2018). Silencing of miR156 confers enhanced resistance to brown planthopper in rice. Planta.

[23] Zhang LL, Li Y, Zheng YP, Wang H, Yang X, Chen JF (2020). Expressing a target mimic of miR156fhl-3p enhances Rice Blast Disease Resistance without Yield Penalty by improving *SPL14* expression. Frontiers in Genetics.

[24] Cao JY, Xu YP, Zhao L, Li SS, Cai XZ (2016). Tight regulation of the interaction between *Brassica napus* and *Sclerotinia sclerotiorum* at the microRNA level. Plant Molecular Biology.

[25] Regmi R, Newman TE, Kamphuis LG, Derbyshire MC (2020). Combined degradome and replicated small RNA sequencing identifies *Brassica napus* small RNAs responsive to infection by a necrotrophic pathogen. BMC Plant Biology.

[26] Li Y, Tong Y, He X, Zhu Y, Li T, Lin X (2022). The rice miR171b-*SCL6*-*IIs* module controls blast resistance, grain yield, and flowering. The Crop Journal.

[27] Liu X, Liu S, Chen X, Prasanna BM, Ni Z, Li X (2022). Maize miR167-*ARF3*/*30*-*polyamine oxidase 1* module-regulated H_2__2_ production confers resistance to maize chlorotic mottle virus. Plant Physiology.

[28] Dangl JL, Horvath DM, Staskawicz BJ (2013). Pivoting the plant immune system from dissection to deployment. Science.

[29] Cesari S, Thilliez G, Ribot C, Chalvon V, Michel C, Jauneau A (2013). The rice resistance protein pair RGA4/RGA5 recognizes the *Magnaporthe oryzae* effectors AVR-Pia and AVR1-CO39 by direct binding. The Plant Cell.

[30] Zipfel C, Kunze G, Chinchilla D, Caniard A, Jones JD, Boller T (2006). Perception of the bacterial PAMP EF-Tu by the receptor EFR restricts *Agrobacterium*-mediated transformation. Cell.

[31] Chinchilla D, Zipfel C, Robatzek S, Kemmerling B, Nurnberger T, Jones JD (2007). A flagellin-induced complex of the receptor FLS2 and BAK1 initiates plant defence. Nature.

[32] Palm-Forster MAT, Eschen-Lippold L, Uhrig J, Scheel D, Lee J (2017). A novel family of proline/serine-rich proteins, which are phospho-targets of stress-related mitogen-activated protein kinases, differentially regulates growth and pathogen defense in *Arabidopsis thaliana*. Plant Molecular Biology.

[33] Yan H, Zhao Y, Shi H, Li J, Wang Y, Tang D (2018). BRASSINOSTEROID-SIGNALING KINASE1 phosphorylates MAPKKK5 to regulate immunity in *Arabidopsis*. Plant Physiology.

[34] Wang J, Grubb LE, Wang J, Liang X, Li L, Gao C (2018). A Regulatory Module Controlling Homeostasis of a Plant Immune kinase. Molecular Cell.

[35] Verma V, Ravindran P, Kumar PP (2016). Plant hormone-mediated regulation of stress responses. BMC Plant Biology.

[36] Jalali BL, Bhargava S, Kamble A (2006). Signal transduction and transcriptional regulation of plant defence responses. Journal of Phytopathology.

[37] Qiu JL, Fiil BK, Petersen K, Nielsen HB, Botanga CJ, Thorgrimsen S (2008). *Arabidopsis MAP kinase 4* regulates gene expression through transcription factor release in the nucleus. The EMBO Journal.

[38] Seo PJ, Kim MJ, Park JY, Kim SY, Jeon J, Lee YH (2010). Cold activation of a plasma membrane-tethered NAC transcription factor induces a pathogen resistance response in *Arabidopsis*. Plant J.

[39] Tsuda K, Somssich IE (2015). Transcriptional networks in plant immunity. New Phytologist.

[40] Jensen MK, Rung JH, Gregersen PL, Gjetting T, Fuglsang AT, Hansen M (2007). The *HvNAC6* transcription factor: a positive regulator of penetration resistance in barley and *Arabidopsis*. Plant Molecular Biology.

[41] Chen LG, Zhang LP, Li DB, Wang F, Yu DQ (2013). *WRKY8* transcription factor functions in the TMV-cg defense response by mediating both abscisic acid and ethylene signaling in *Arabidopsis*. Proceedings of the National Academy of Sciences of the United States of America.

[42] Yamaguchi A, Wu MF, Yang L, Wu G, Poethig RS, Wagner D (2009). The microRNA-regulated SBP-Box transcription factor SPL3 is a direct upstream activator of LEAFY, FRUITFULL, and APETALA1. Developmental Cell.

[43] Kim JJ, Lee JH, Kim W, Jung HS, Huijser P, Ahn JH (2012). The microRNA156-*SQUAMOSA PROMOTER BINDING PROTEIN-LIKE3* module regulates ambient temperature-responsive flowering via *FLOWERING LOCUS T* in *Arabidopsis*. Plant physiology.

[44] Feyissa BA, Amyot L, Nasrollahi V, Papadopoulos Y, Kohalmi SE, Hannoufa A (2021). Involvement of the miR156/SPL module in flooding response in *Medicago sativa*. Scientific Reports.

[45] Wei L, Jian H, Lu K, Filardo F, Yin N, Liu L (2016). Genome-wide association analysis and differential expression analysis of resistance to *Sclerotinia* stem rot in *Brassica napus*. Plant Biotechnology Journal.

[46] Franco-Zorrilla JM, Valli A, Todesco M, Mateos I, Puga MI, Rubio-Somoza I (2007). Target mimicry provides a new mechanism for regulation of microRNA activity. Nature Genetics.

[47] Clough SJ, Bent AF (1998). Floral dip: a simplified method for *Agrobacterium*-mediated transformation of *Arabidopsis thaliana*. The Plant Journal.

[48] Ding Y, Mei J, Chai Y, Yang W, Mao Y, Yan B (2020). *Sclerotinia sclerotiorum* utilizes host-derived copper for ROS detoxification and infection. PLOS Pathogens.

[49] Chalhoub B, Denoeud F, Liu SY, Parkin IAP, Tang HB, Wang XY (2014). Early allopolyploid evolution in the post-neolithic *Brassica napus* oilseed genome. Science.

[50] Kozomara A, Griffiths-Jones S (2014). miRBase: annotating high confidence microRNAs using deep sequencing data. Nucleic Acids Research.

[51] Dai XB, Zhuang ZH, Zhao PXC (2018). psRNATarget: a plant small RNA target analysis server (2017 release). Nucleic Acids Research.

[52] de Oliveira MV, Xu G, Li B, de Souza Vespoli L, Meng X, Chen X (2016). Specific control of *Arabidopsis BAK1*/*SERK4*-regulated cell death by protein glycosylation. Nature Plants.

[53] Livak KJ, Schmittgen TD (2001). Analysis of relative gene expression data using real-time quantitative PCR and the 2 ^-∆∆CT^ method. Methods.

