# Supplementary figures and images for: Screening of microRNAs and target genes involved in Sclerotinia sclerotiorum (Lib.) infection in Brassica napus L
Source: BMC Plant Biol. 2023 Oct 9;23:479. doi: 10.1186/s12870-023-04501-7 (PMC10561407; doi:10.1186/s12870-023-04501-7)

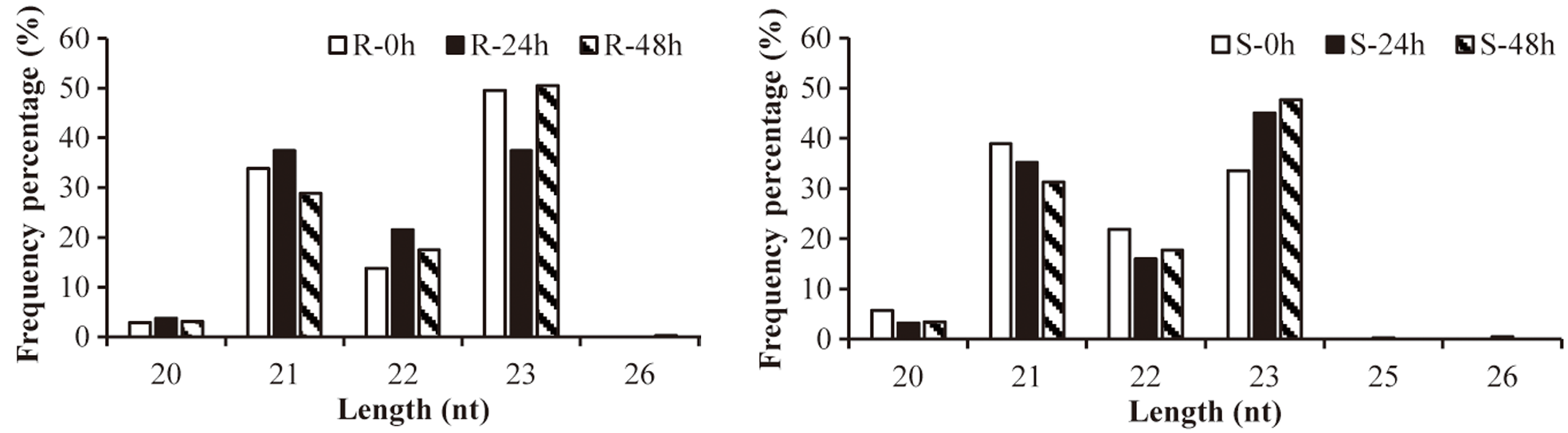

Supplement: Supplementary file 1 — Supplementary Material 1: Fig. S1 Length distribution of novel RNAs predicted in this study [file 12870_2023_4501_MOESM1_ESM.png]

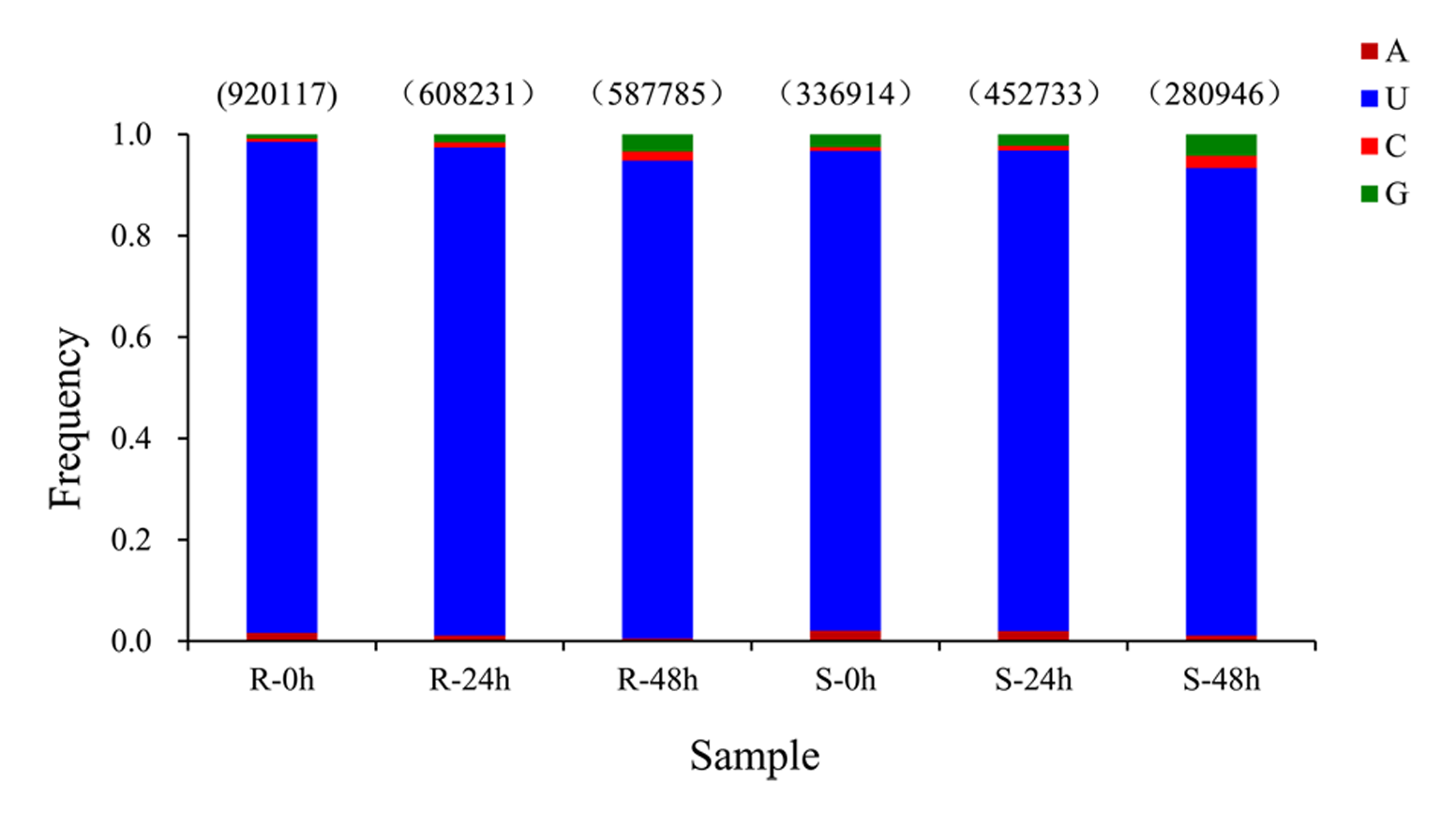

Supplement: Supplementary file 2 — Supplementary Material 2: Fig. S2 First base bias of 21-nt novel miRNAs. The y-axis represents the frequency of nucleotides and the x-axis represents different libraries. Four different colours in the bars represent the four nucleotides [file 12870_2023_4501_MOESM2_ESM.png]

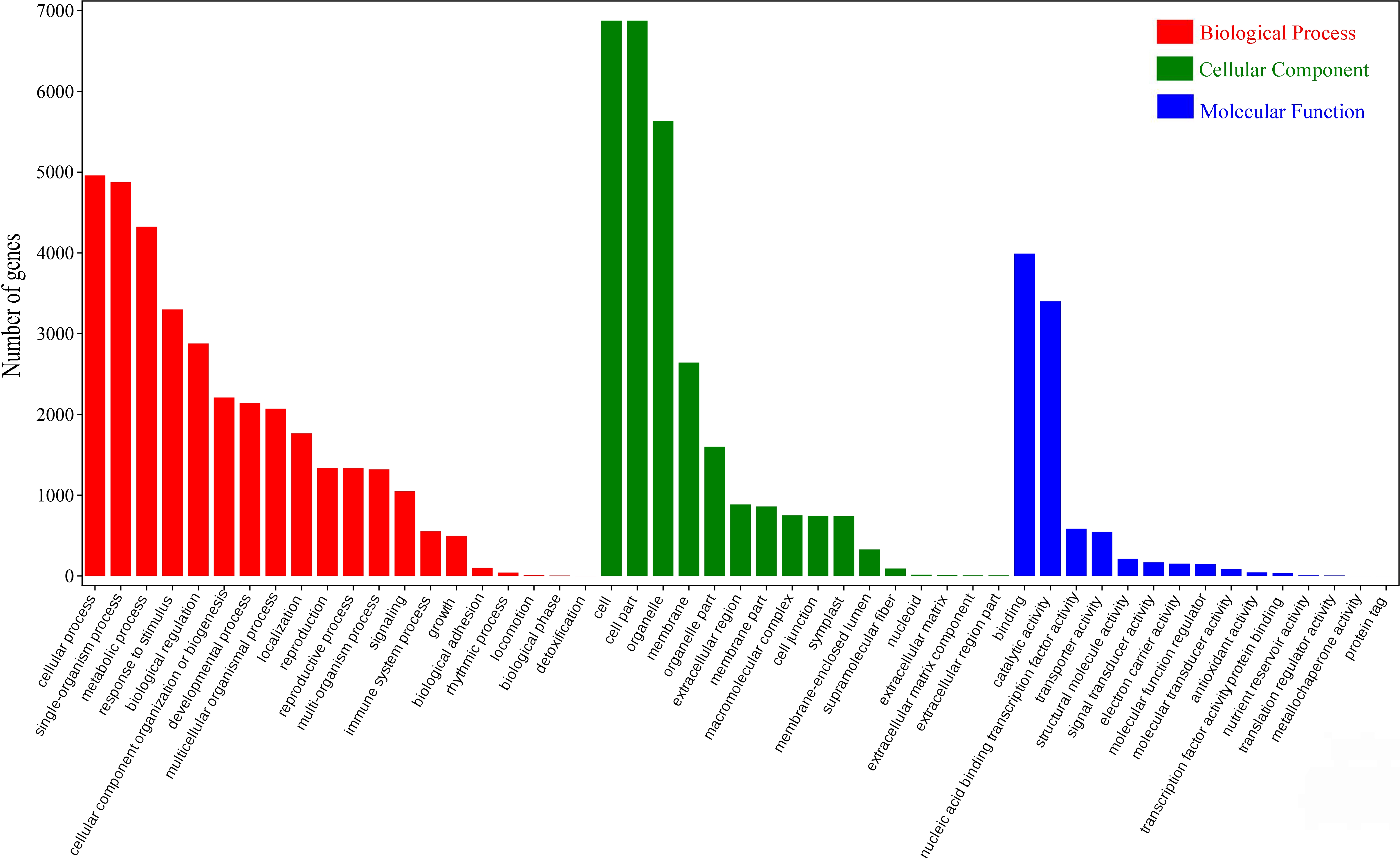

Supplement: Supplementary file 3 — Supplementary Material 3: Fig. S3 GO annotation of all target genes. The y-axis (left) represents the percentages of genes identified in this study, and the y-axis (right) represents the actual gene number. The genes were annotated in three main categories [file 12870_2023_4501_MOESM3_ESM.png]

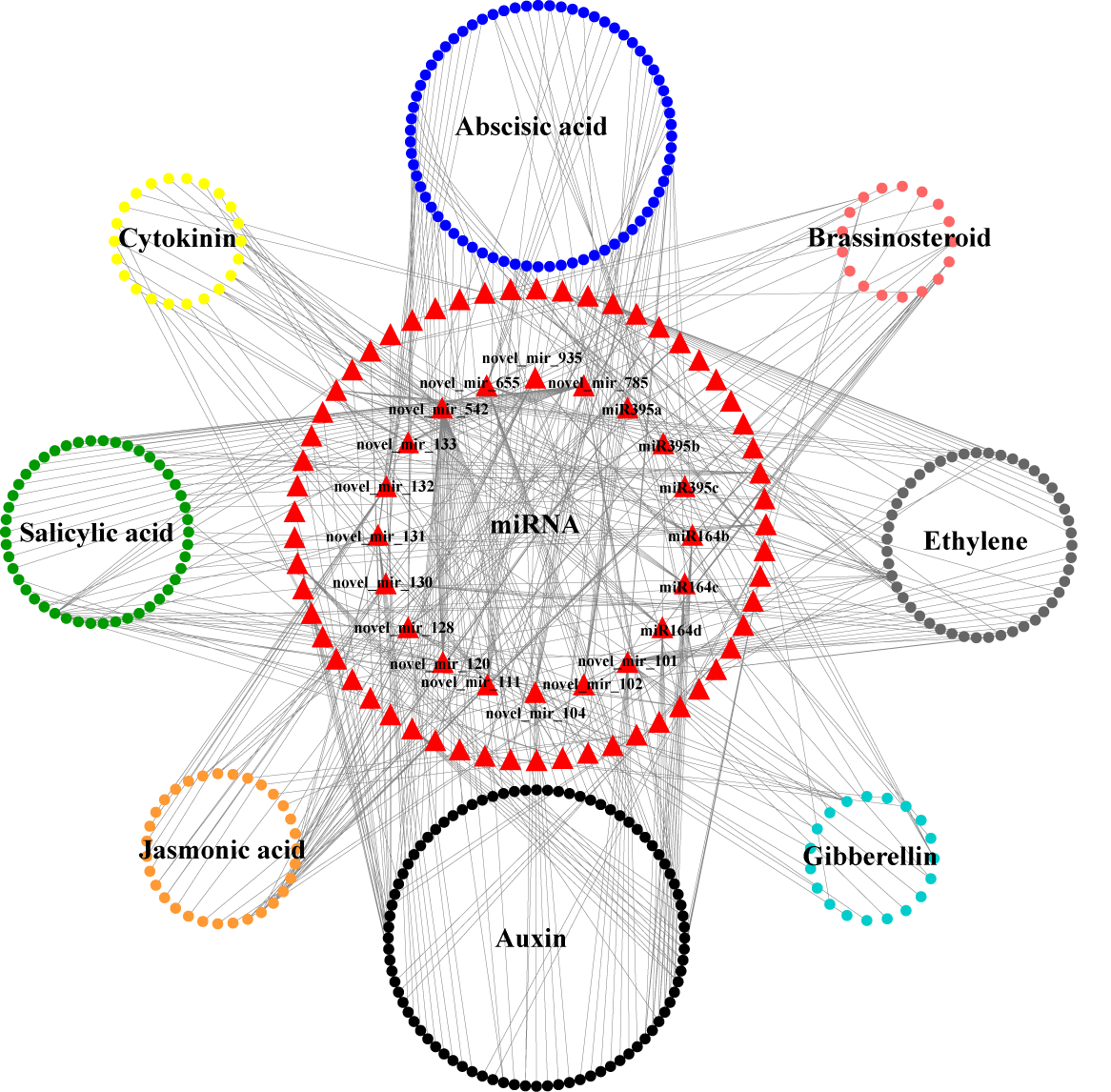

Supplement: Supplementary file 4 — Supplementary Material 4: Fig. S4 Hormone-related genes targeted by miRNAs were shown to be involved in regulatory network mechanisms of miRNA-target module response to S. sclerotiorum infection in B. napus. Red triangle means miRNAs; solid circle means target genes involved in hormone metabolism and signal transduction; black circle means auxin-related genes; blue circle means abscisic acid-related genes; grey circle means ethylene-related genes; green circle means salicylic acid-related genes; yellow circle means cytokinin-related genes; red circle means brassinosteroid; light blue means gibberellin-related genes; orange yellow means jasmonic acid-related genes [file 12870_2023_4501_MOESM4_ESM.png]

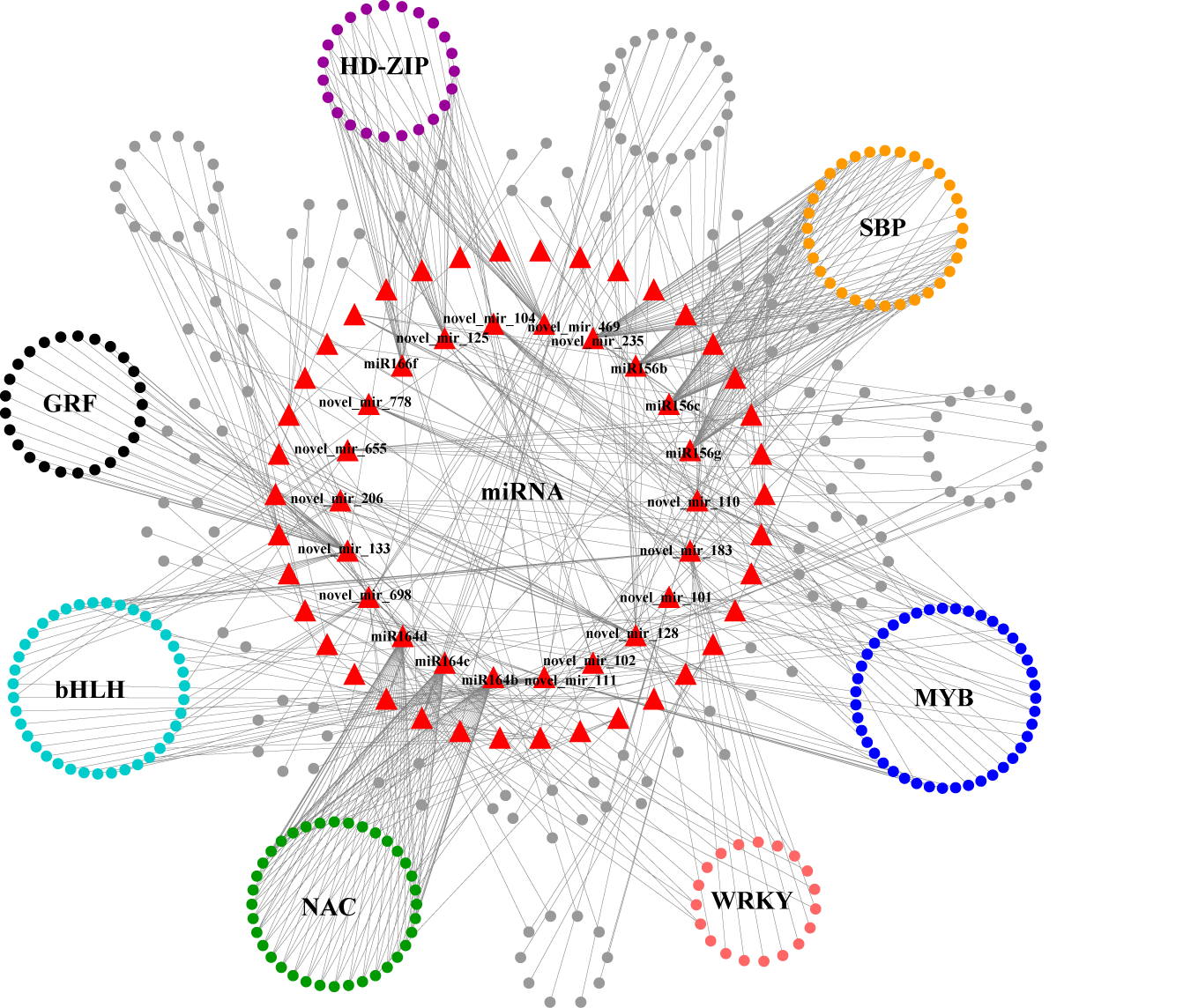

Supplement: Supplementary file 5 — Supplementary Material 5: Fig. S5 TF genes targeted by miRNAs were found to be involved in regulatory network mechanisms of miRNA-target module response to S. sclerotiorum infection in B. napus. Red triangle means miRNAs; solid circle means target genes encoding TF genes; blue circle means MYB genes; green circle means NAC genes; light blue means bHLH genes; black circle means GRF genes; purple means HD-ZIP genes; yellow means SBP genes; pink circle means WRKY genes [file 12870_2023_4501_MOESM5_ESM.png]

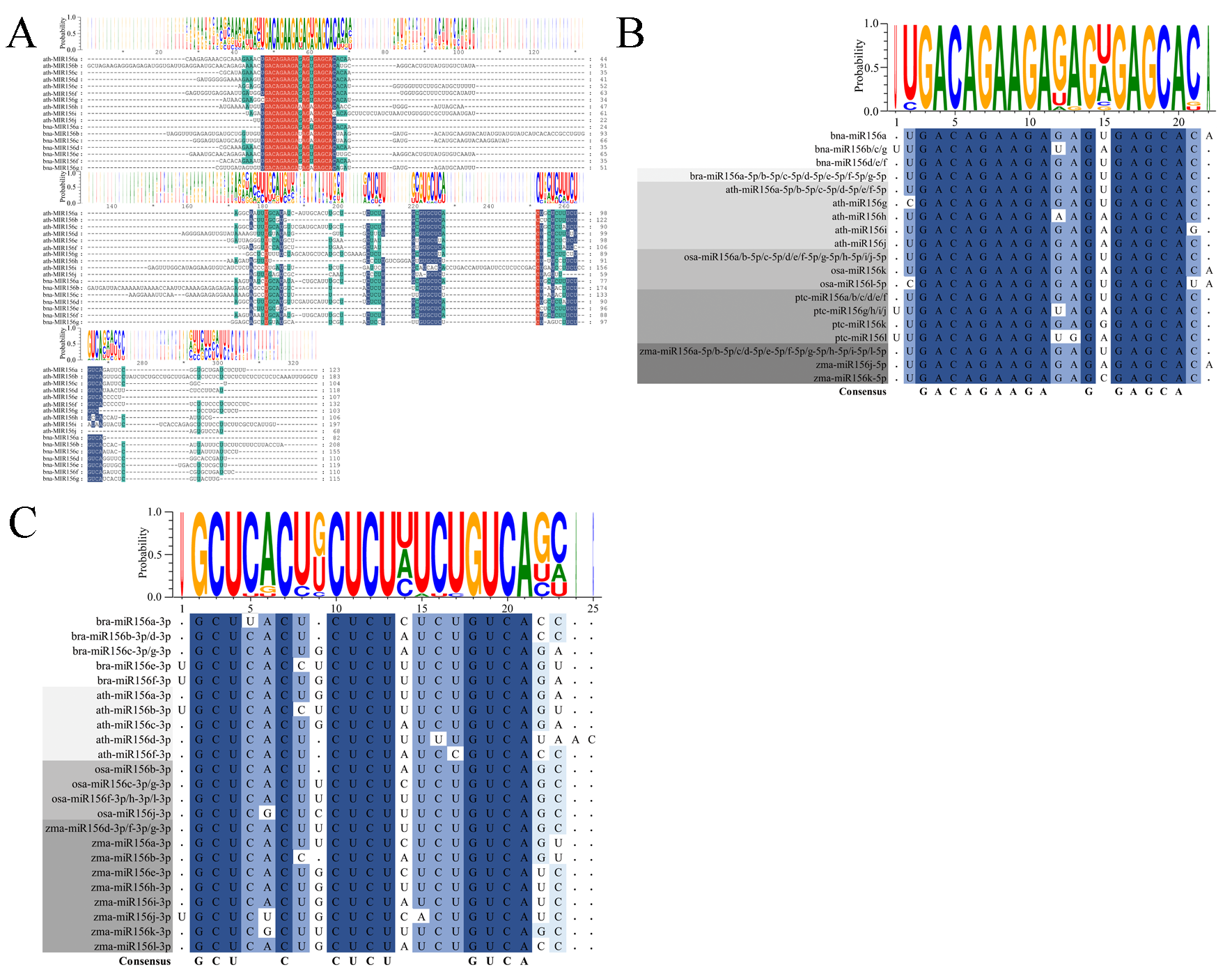

Supplement: Supplementary file 6 — Supplementary Material 6: Fig. S6 Multiple sequence alignment result analysis. (A) Multiple sequence alignment of precursor sequences of miR156 family members from Arabidopsis and rapeseed. (B-C) Multiple sequence alignment of mature sequences of miR156 family members from several species. bna means Brassica napus; bra means Brassica rapa; ath means Arabidopsis thaliana; osa means Oryza sativa; ptc means Populus trichocarpa; zma means Zea mays [file 12870_2023_4501_MOESM6_ESM.png]

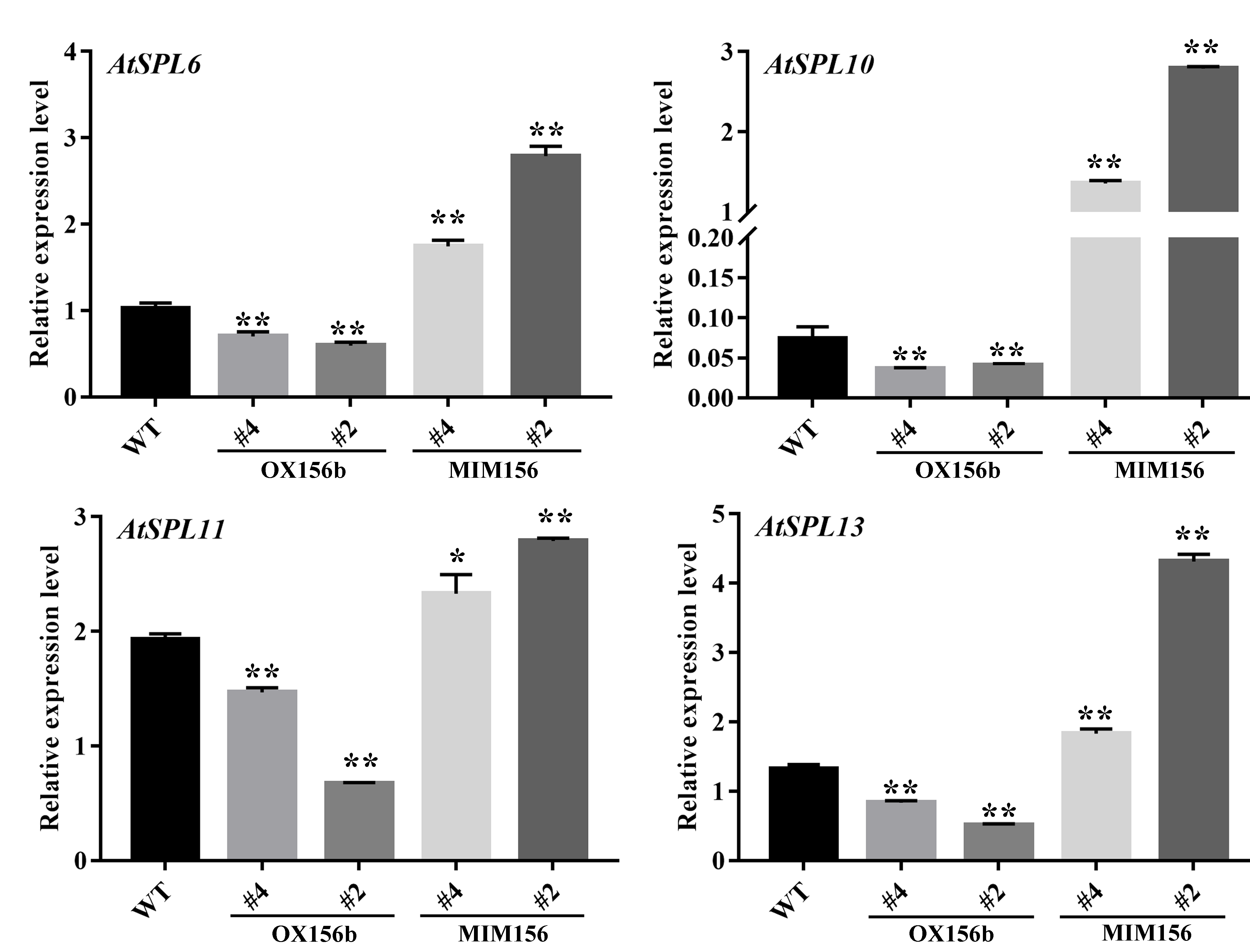

Supplement: Supplementary file 7 — Supplementary Material 7: Fig. S7 Expression levels of the miR156 target genes AtSPL6, AtSPL10, AtSPL11, and AtSPL13 in WT, MIM156, and OX156b leaves from 4-week-old Arabidopsis plants. Values are the means ± SDs from three replicates. Data are the means ± SDs from three independent experiments. The significant differences from WT are indicated (Student’s t test: **. P < 0.01) [file 12870_2023_4501_MOESM7_ESM.png]

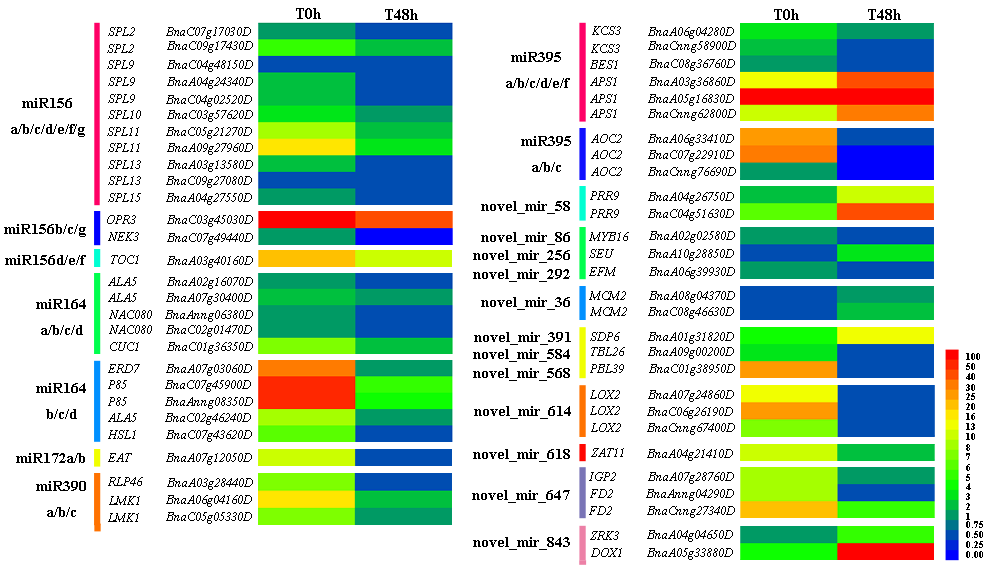

Supplement: Supplementary file 8 — Supplementary Material 8: Fig. S8 Heatmap of 56 predicted target genes for DEMs in resistant B. napus after inoculation with S. sclerotiorum [file 12870_2023_4501_MOESM8_ESM.png]
